# Supplementary figures and images for: Telomeric DNA damage response mediates neurotoxicity of Aβ42 oligomers in Alzheimer’s disease
Source: EMBO J. 2025 Sep 21;44(21):6078–111. doi: 10.1038/s44318-025-00521-1 (PMC12583505; doi:10.1038/s44318-025-00521-1)

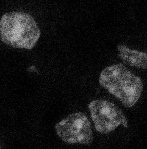

Supplement: Supplementary file 2 — Source data Fig. 1 [file 44318_2025_521_MOESM2_ESM.zip › Fig1/Fig1A/Image Fig 1A/Fig1A_DAPI_MAP2_wt_left_panel.tif]

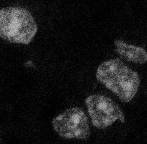

Supplement: Supplementary file 2 — Source data Fig. 1 [file 44318_2025_521_MOESM2_ESM.zip › Fig1/Fig1A/Image Fig 1A/Fig1A_DAPI_gH2AX_wt_left_panel.tif]

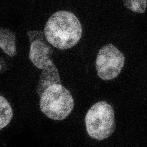

Supplement: Supplementary file 2 — Source data Fig. 1 [file 44318_2025_521_MOESM2_ESM.zip › Fig1/Fig1B/images Fig 1B/Fig1B_DAPI_MAP2_3XTgAD_left_panel.tif]

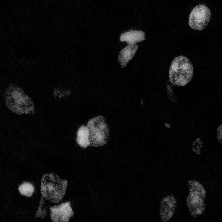

Supplement: Supplementary file 2 — Source data Fig. 1 [file 44318_2025_521_MOESM2_ESM.zip › Fig1/Fig1H/Images Fig1H/Fig1H_DAPI_Map2_wt.tif]

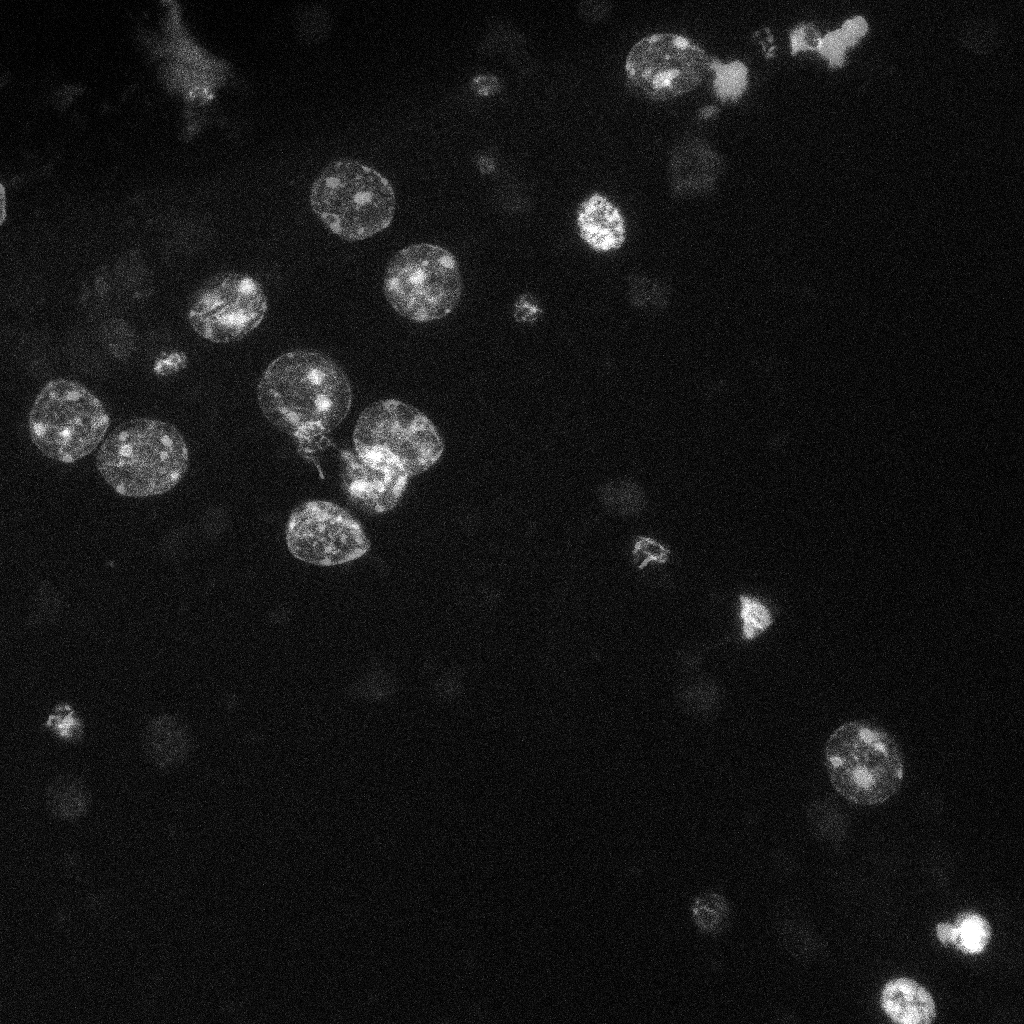

Supplement: Supplementary file 3 — Source data Fig. 2 [file 44318_2025_521_MOESM3_ESM.zip › Fig2/Fig2A/Images_Fig2A/Fig2BAbeta_DAPI_MAP2_right panel.tif]

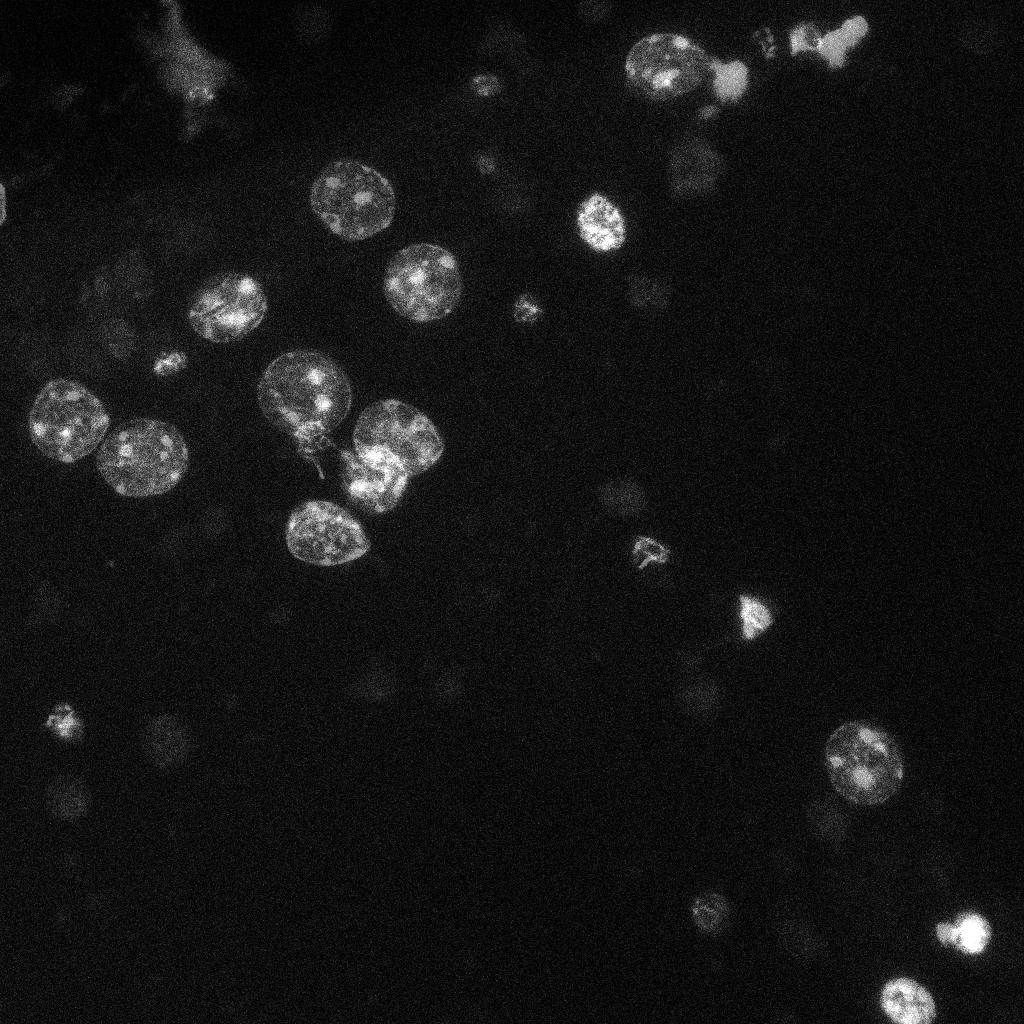

Supplement: Supplementary file 3 — Source data Fig. 2 [file 44318_2025_521_MOESM3_ESM.zip › Fig2/Fig2A/Images_Fig2A/Fig2B_Abeta_DAPI_pATM_right panel.tif]

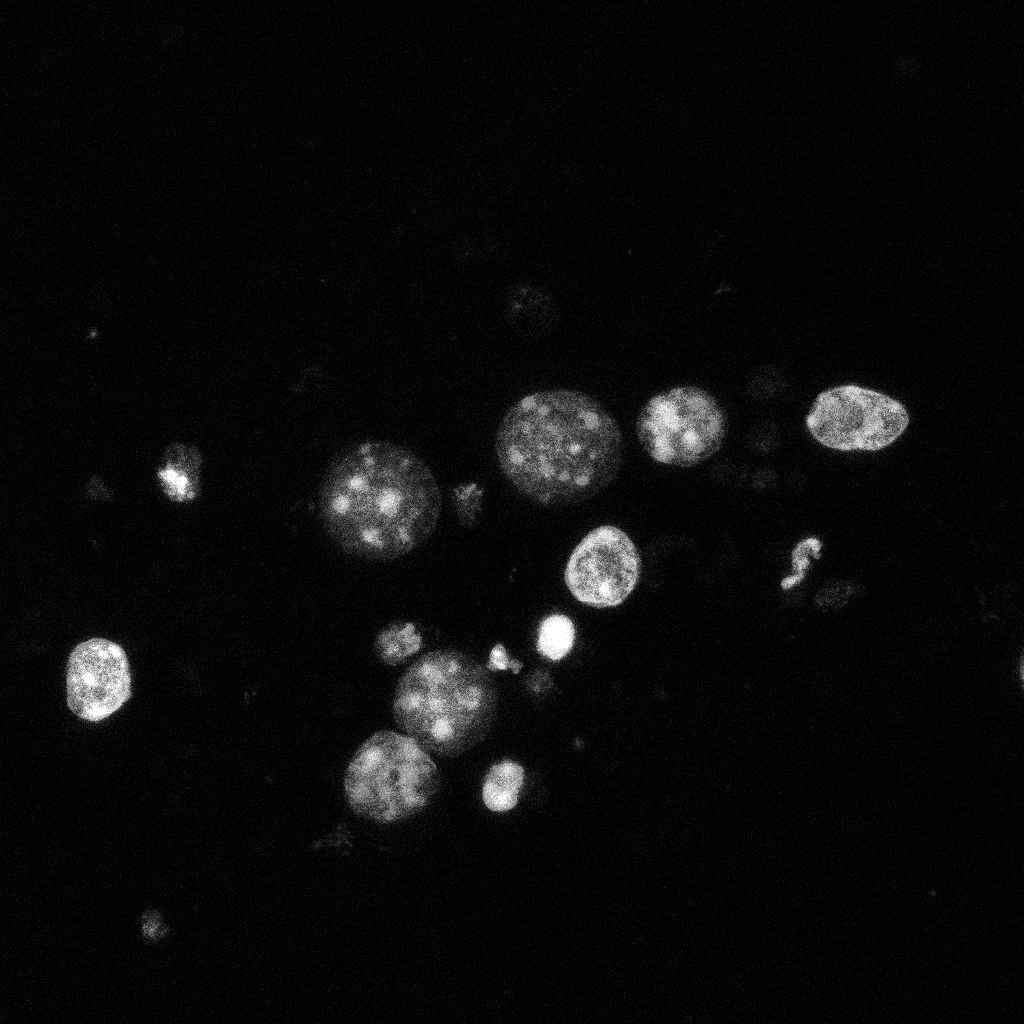

Supplement: Supplementary file 3 — Source data Fig. 2 [file 44318_2025_521_MOESM3_ESM.zip › Fig2/Fig2A/Images_Fig2A/Fig2B_Vehicle_DAPI_pATM_right panel.tif]

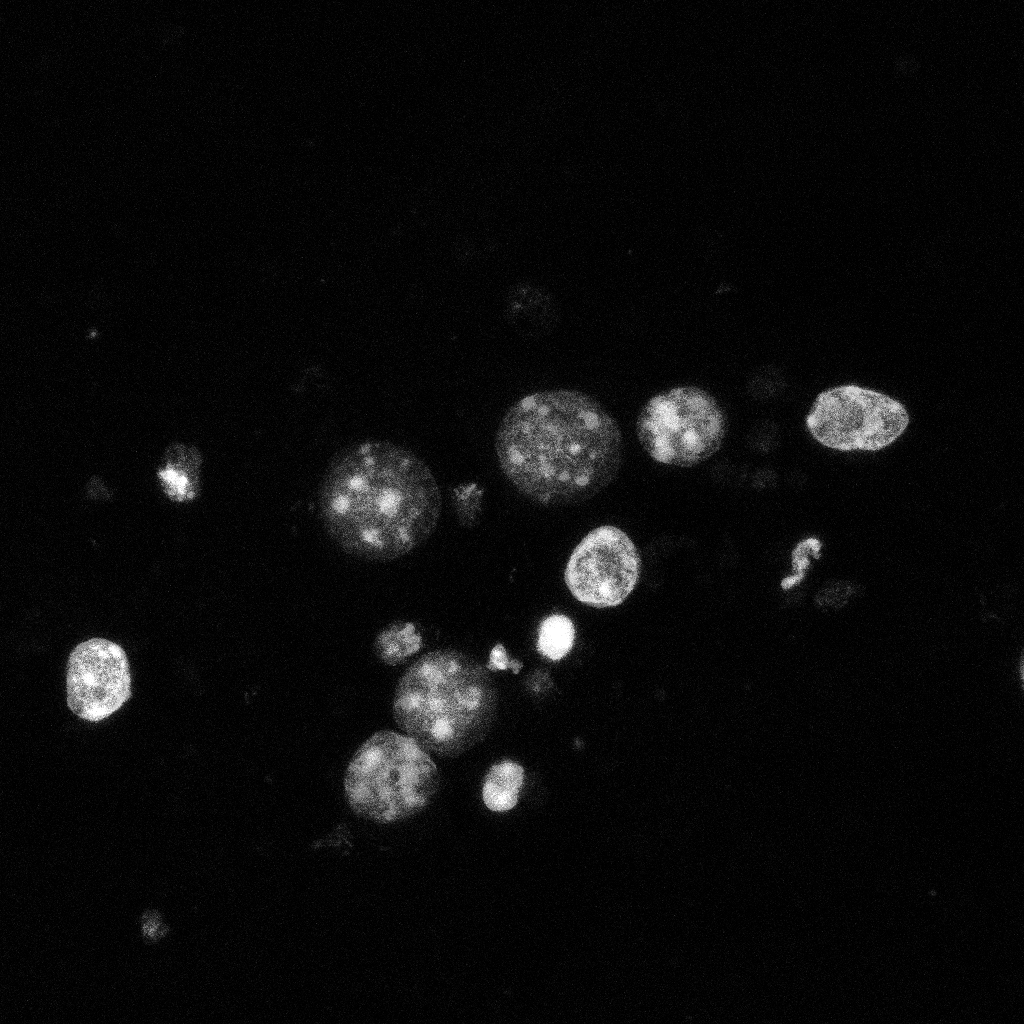

Supplement: Supplementary file 3 — Source data Fig. 2 [file 44318_2025_521_MOESM3_ESM.zip › Fig2/Fig2A/Images_Fig2A/Fig2BVehcile_DAPI_MAP2_right panel.tif]

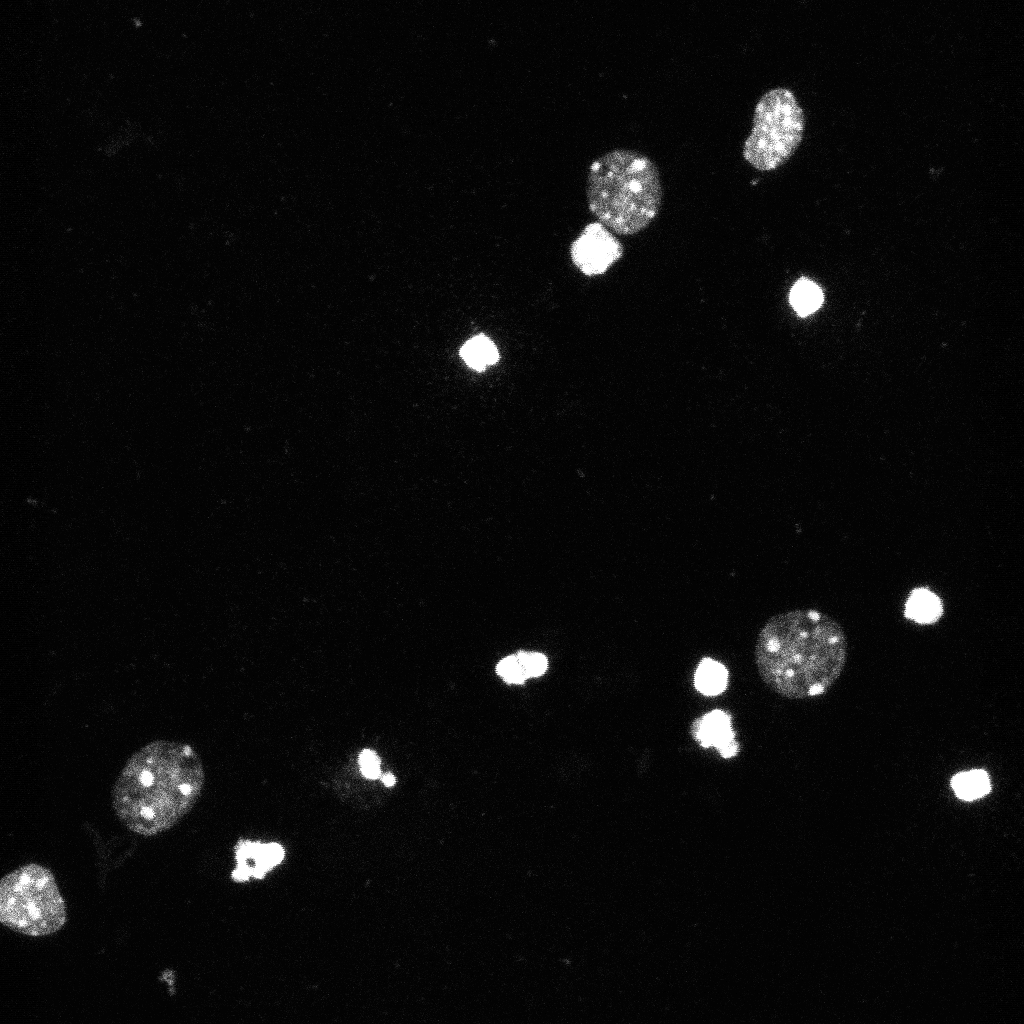

Supplement: Supplementary file 3 — Source data Fig. 2 [file 44318_2025_521_MOESM3_ESM.zip › Fig2/Fig2A/Images_Fig2A/Fig2A_Abeta_DAP_gH2AX_left panel.tif]

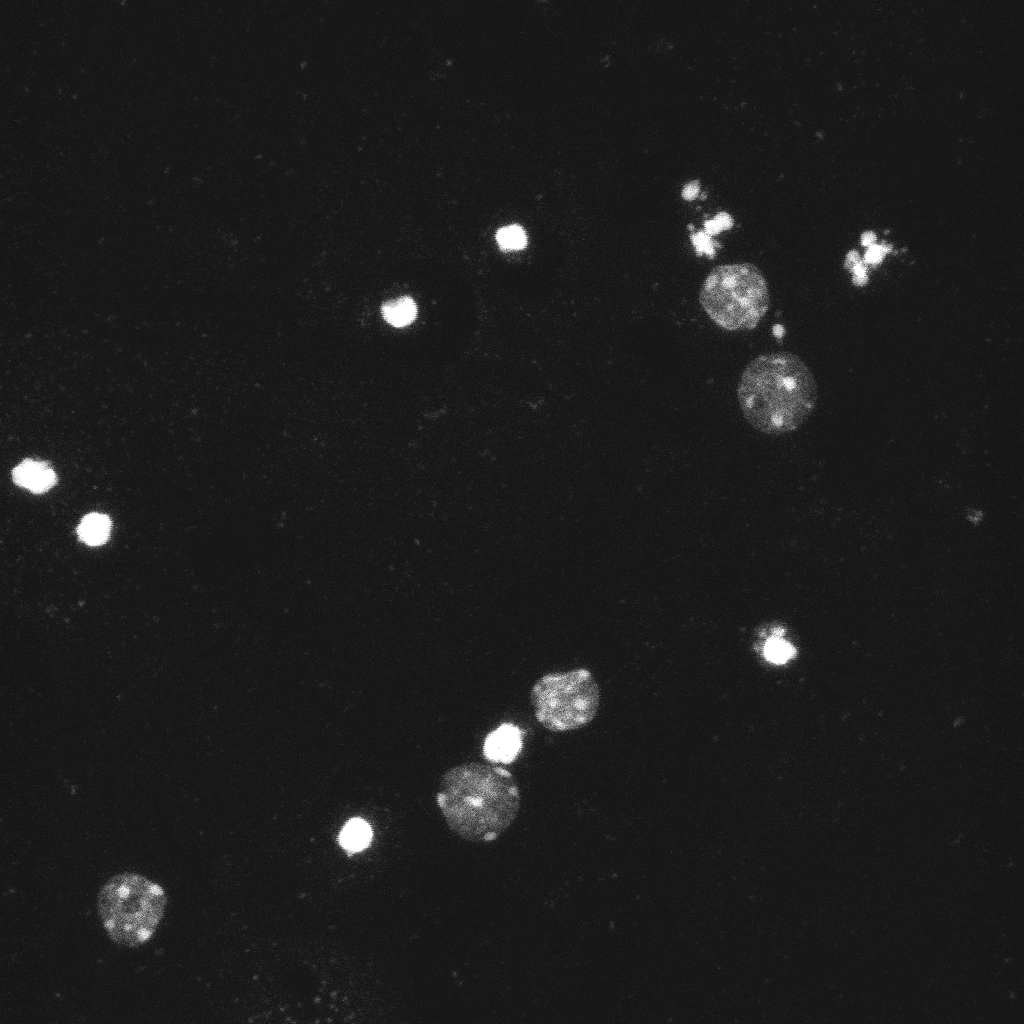

Supplement: Supplementary file 3 — Source data Fig. 2 [file 44318_2025_521_MOESM3_ESM.zip › Fig2/Fig2A/Images_Fig2A/Fig2B_Vehicle_DAPI_gH2AX_left panel.tif]

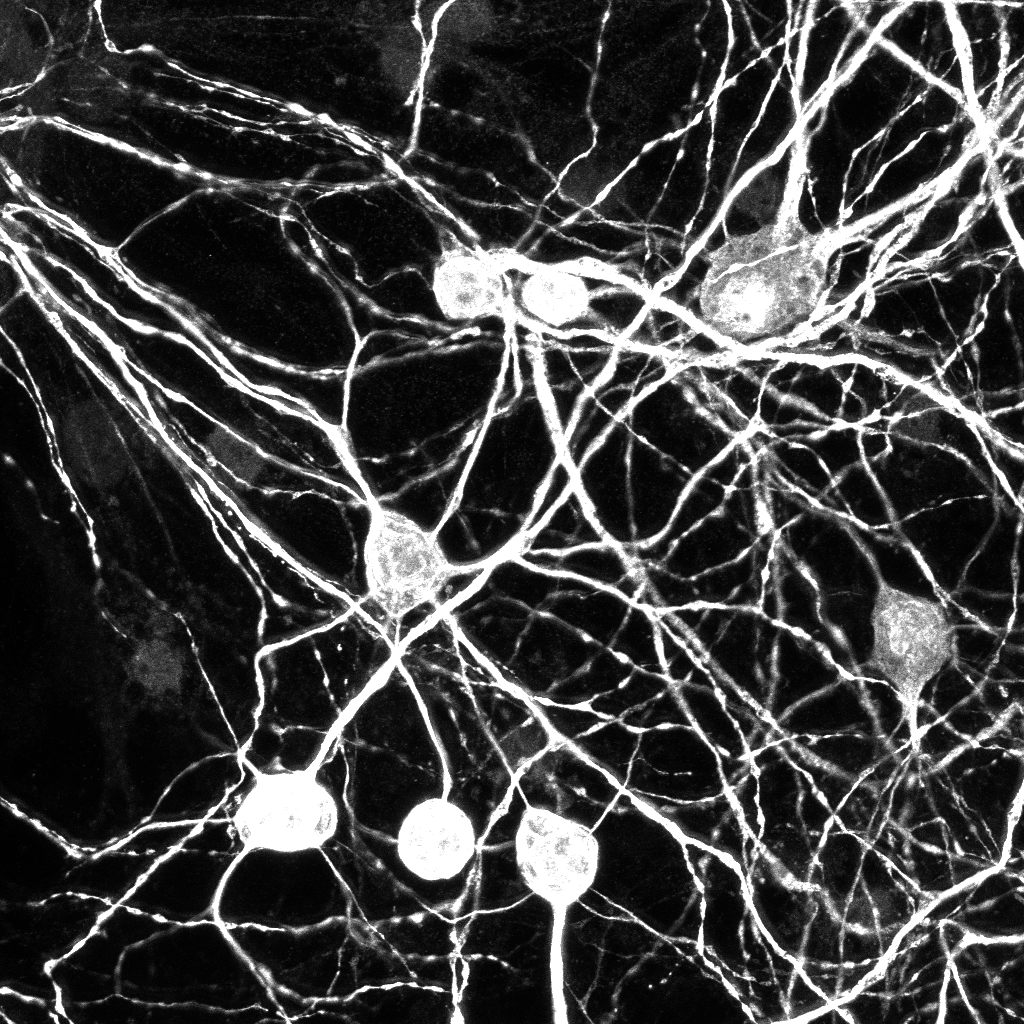

Supplement: Supplementary file 4 — Source data Fig. 3 [file 44318_2025_521_MOESM4_ESM.zip › Fig3/Fig3D/images fig3D/Vhicle MAp2.tif]

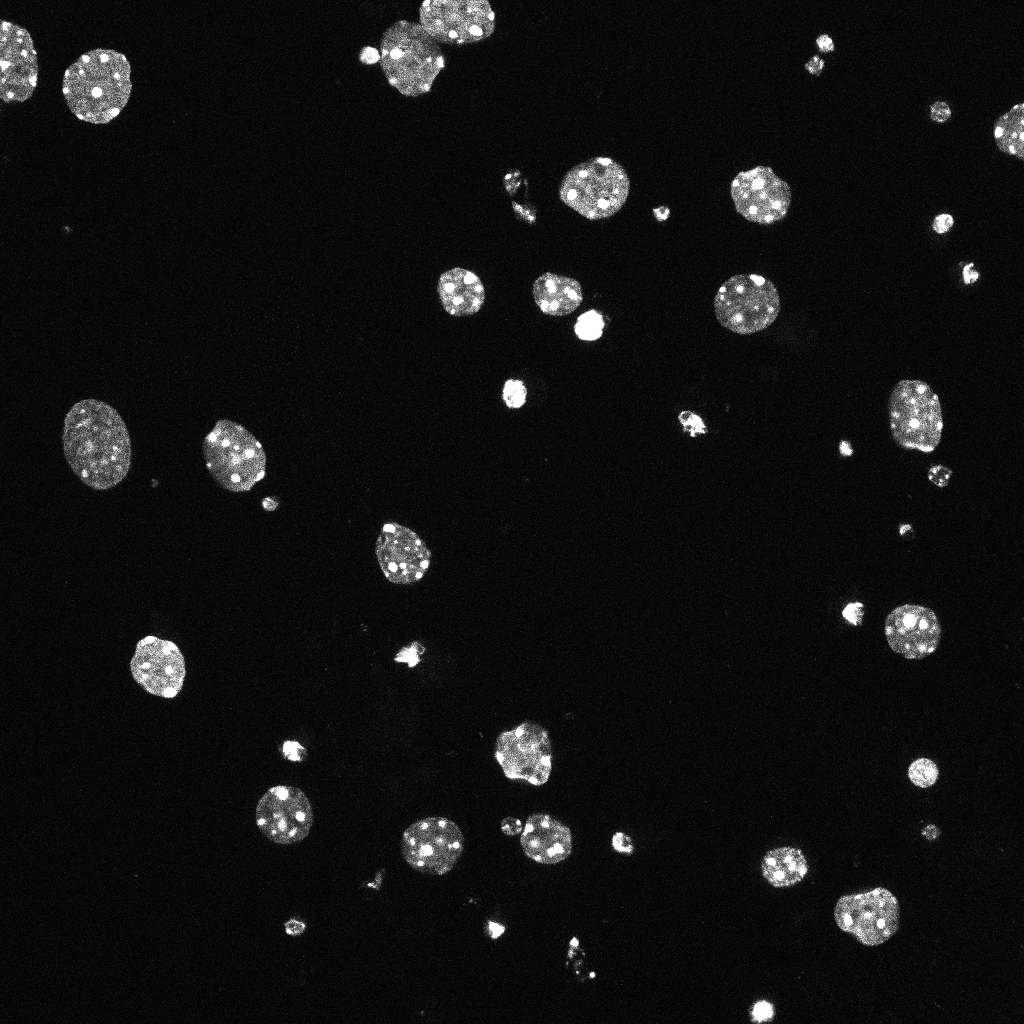

Supplement: Supplementary file 4 — Source data Fig. 3 [file 44318_2025_521_MOESM4_ESM.zip › Fig3/Fig3D/images fig3D/Vheicle DAPI.tif]

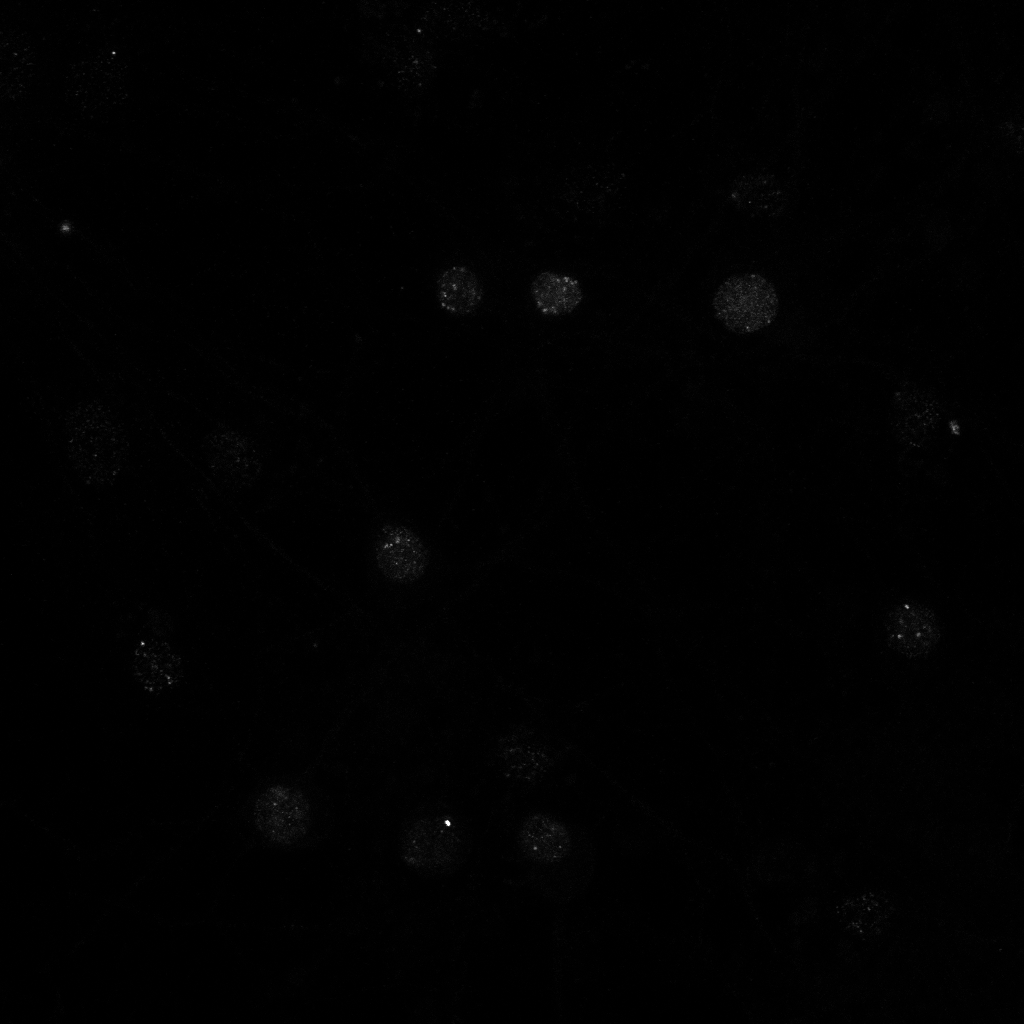

Supplement: Supplementary file 4 — Source data Fig. 3 [file 44318_2025_521_MOESM4_ESM.zip › Fig3/Fig3D/images fig3D/Vheicle gH2AX.tif]

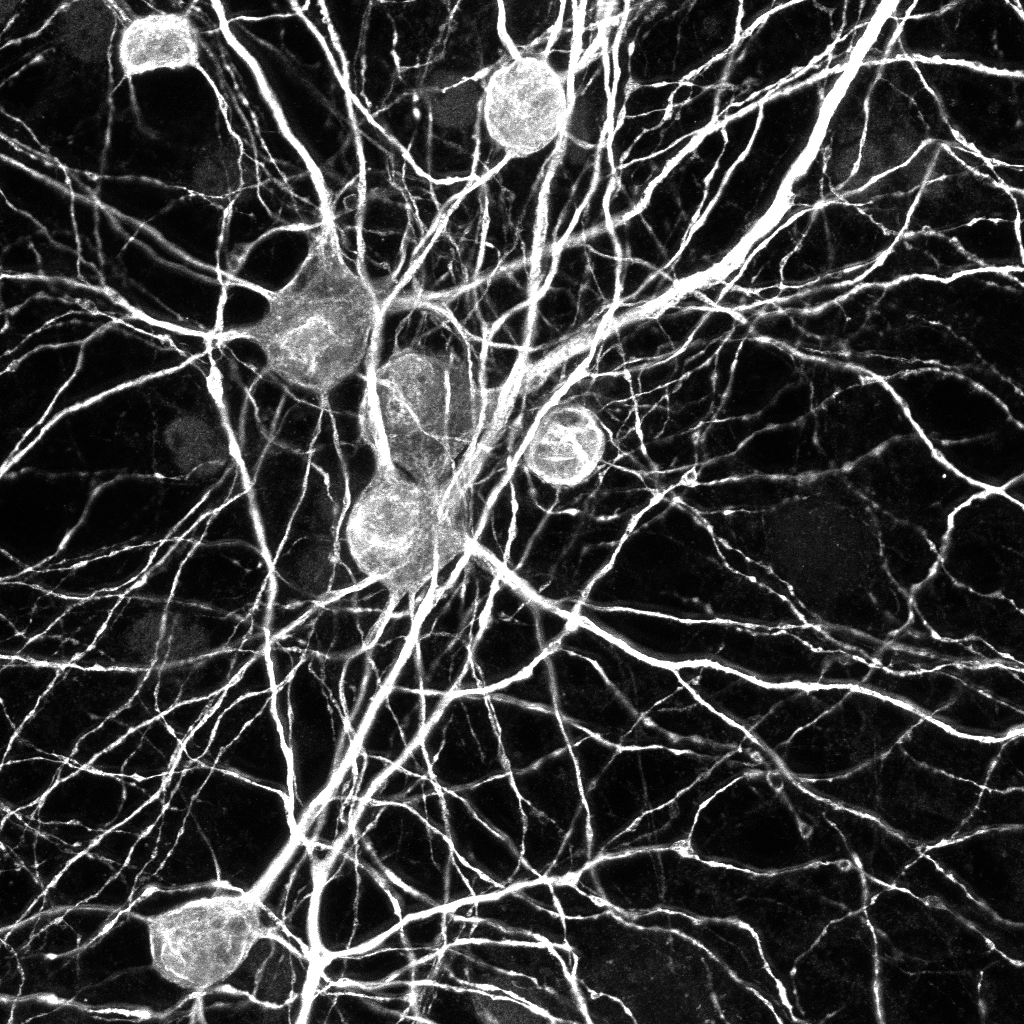

Supplement: Supplementary file 4 — Source data Fig. 3 [file 44318_2025_521_MOESM4_ESM.zip › Fig3/Fig3D/images fig3D/Abeta oligomers MAP2.tif]

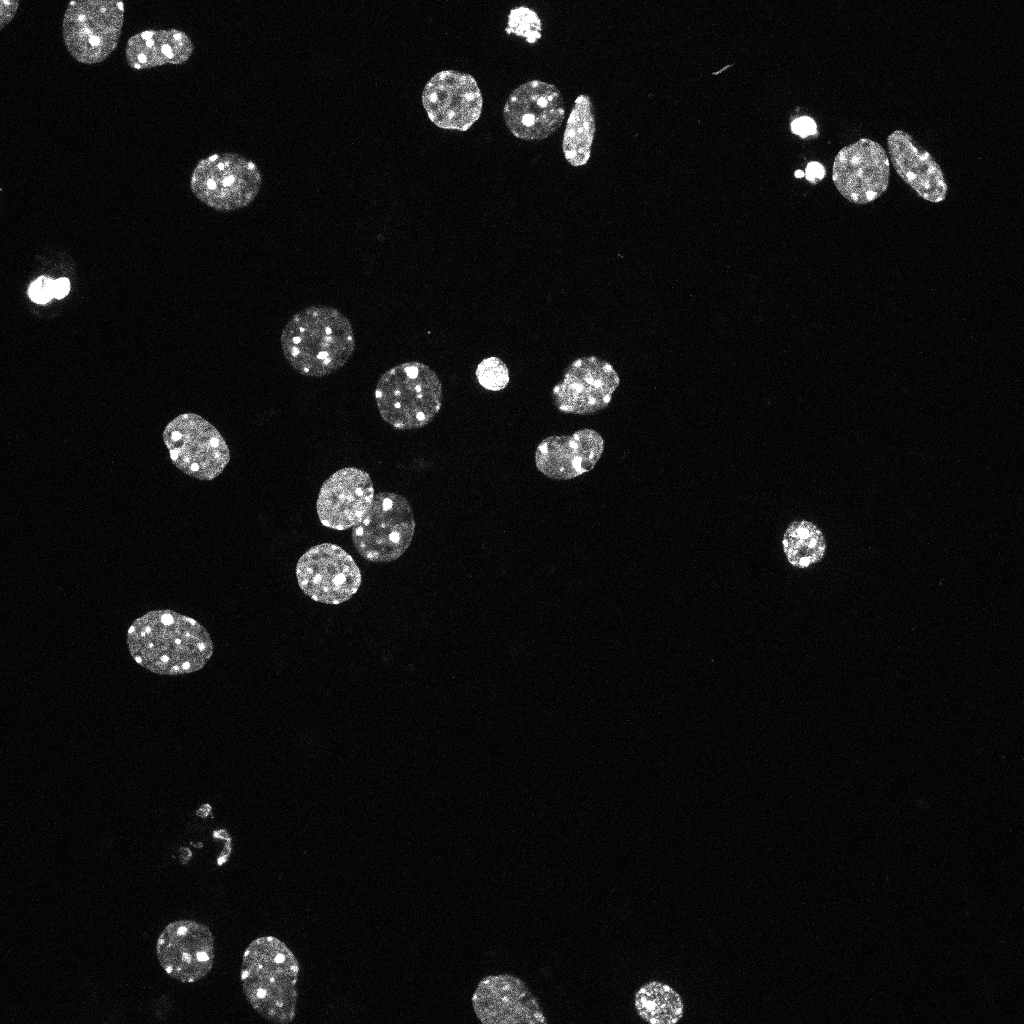

Supplement: Supplementary file 4 — Source data Fig. 3 [file 44318_2025_521_MOESM4_ESM.zip › Fig3/Fig3D/images fig3D/Abeta oligomers DAPI.tif]

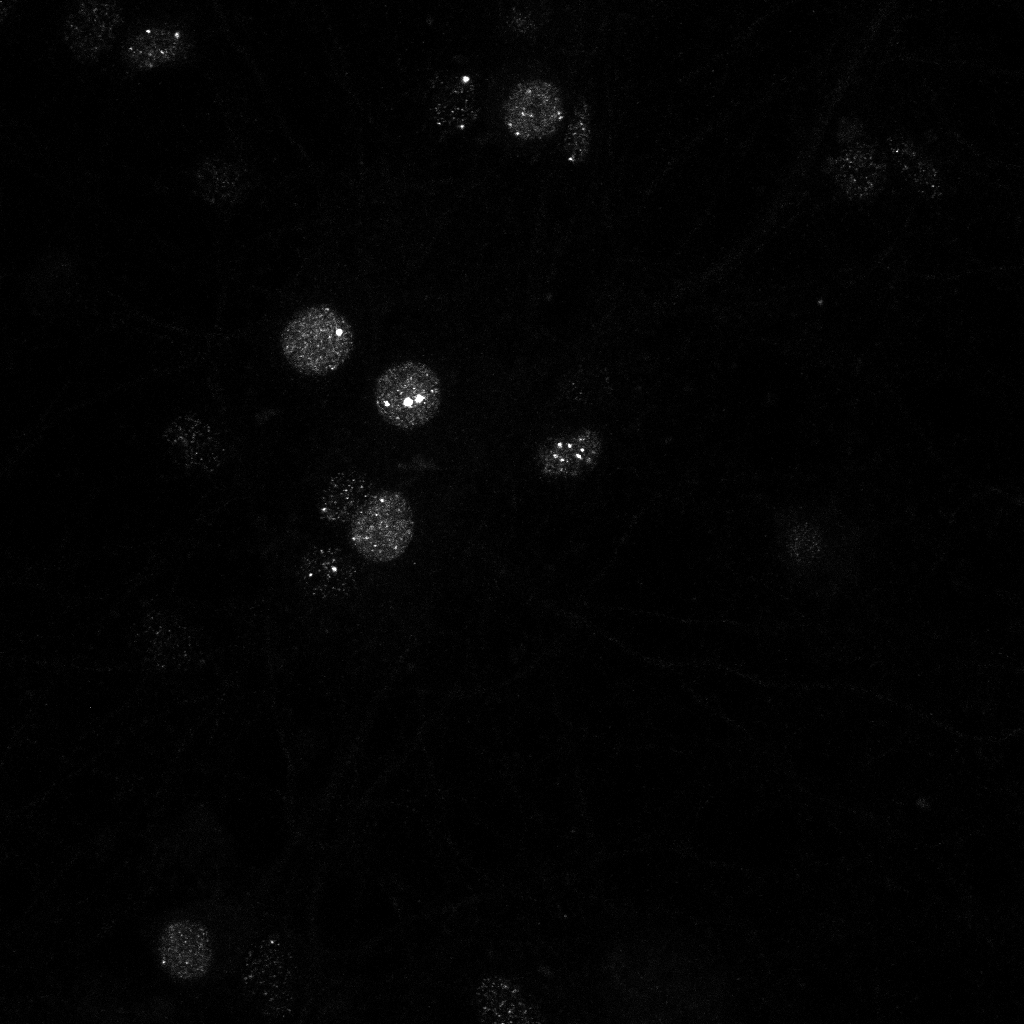

Supplement: Supplementary file 4 — Source data Fig. 3 [file 44318_2025_521_MOESM4_ESM.zip › Fig3/Fig3D/images fig3D/Abeta oligomers gH2AX.tif]

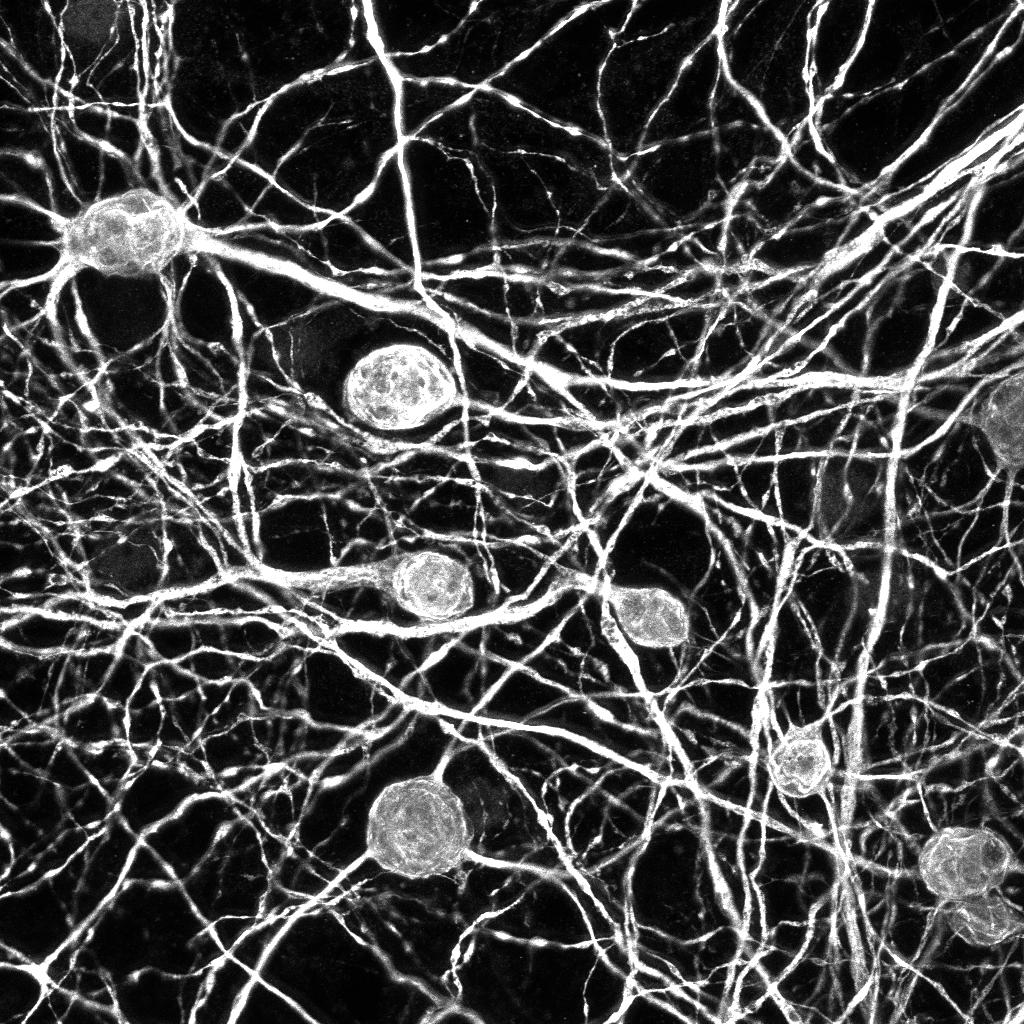

Supplement: Supplementary file 4 — Source data Fig. 3 [file 44318_2025_521_MOESM4_ESM.zip › Fig3/Fig3D/images fig3D/Abeta oligomers_NAC MAP2.tif]

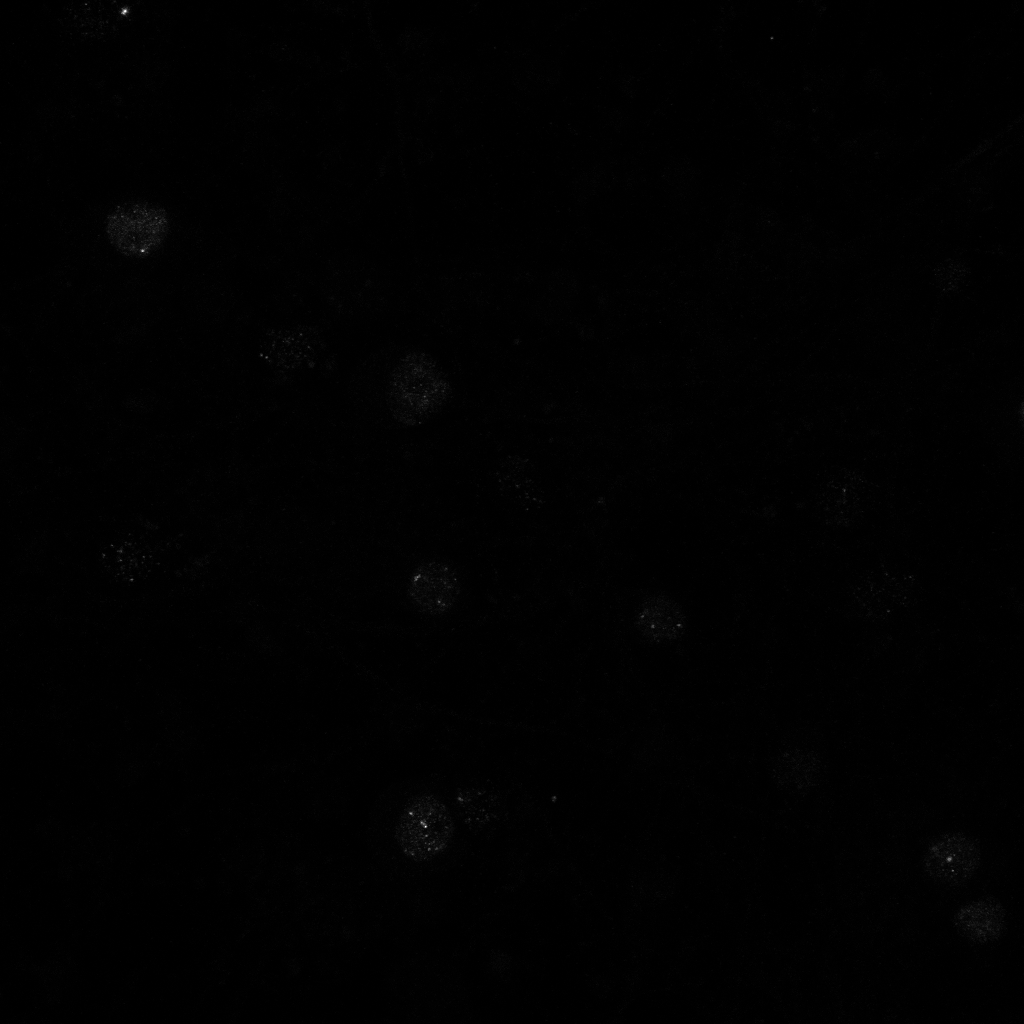

Supplement: Supplementary file 4 — Source data Fig. 3 [file 44318_2025_521_MOESM4_ESM.zip › Fig3/Fig3D/images fig3D/Abeta oligomers_NAC gH2AX.tif]

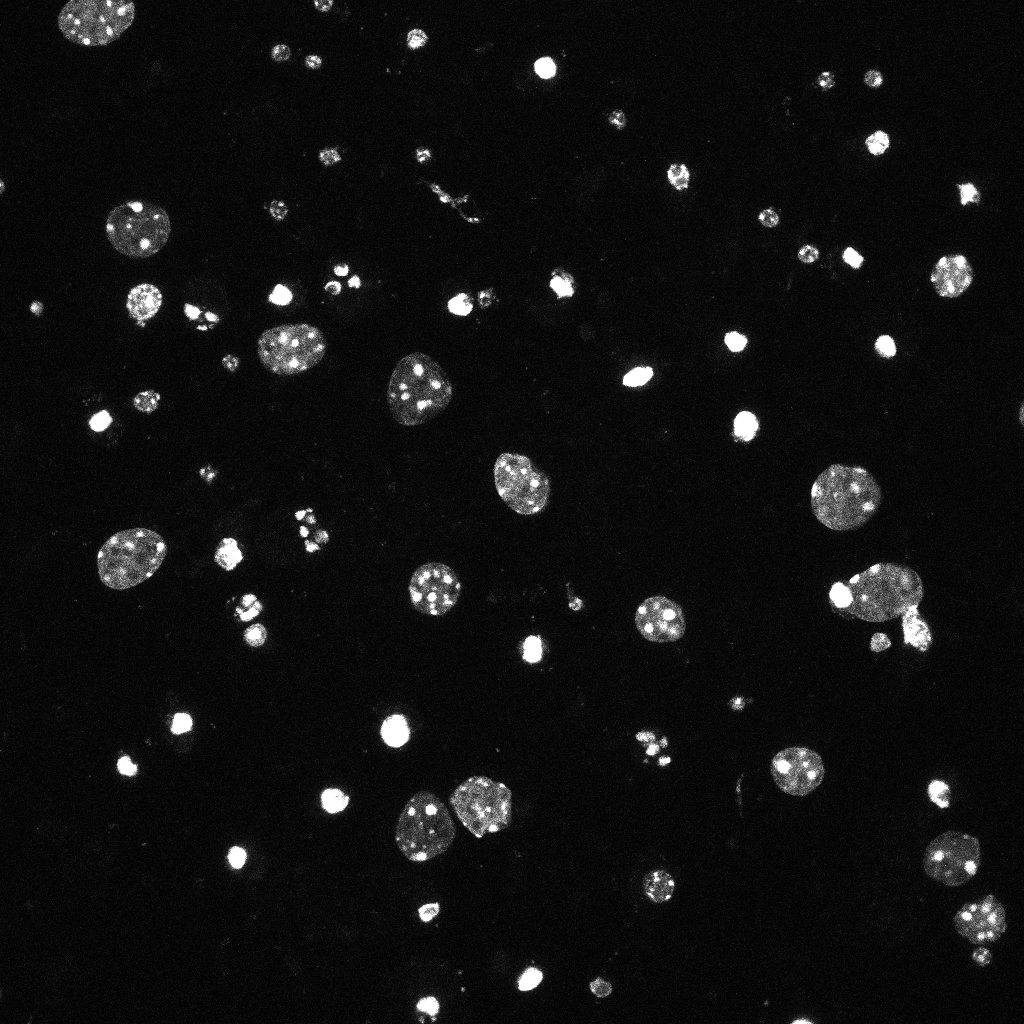

Supplement: Supplementary file 4 — Source data Fig. 3 [file 44318_2025_521_MOESM4_ESM.zip › Fig3/Fig3D/images fig3D/Abeta oligomers_NAC DAPI.tif]

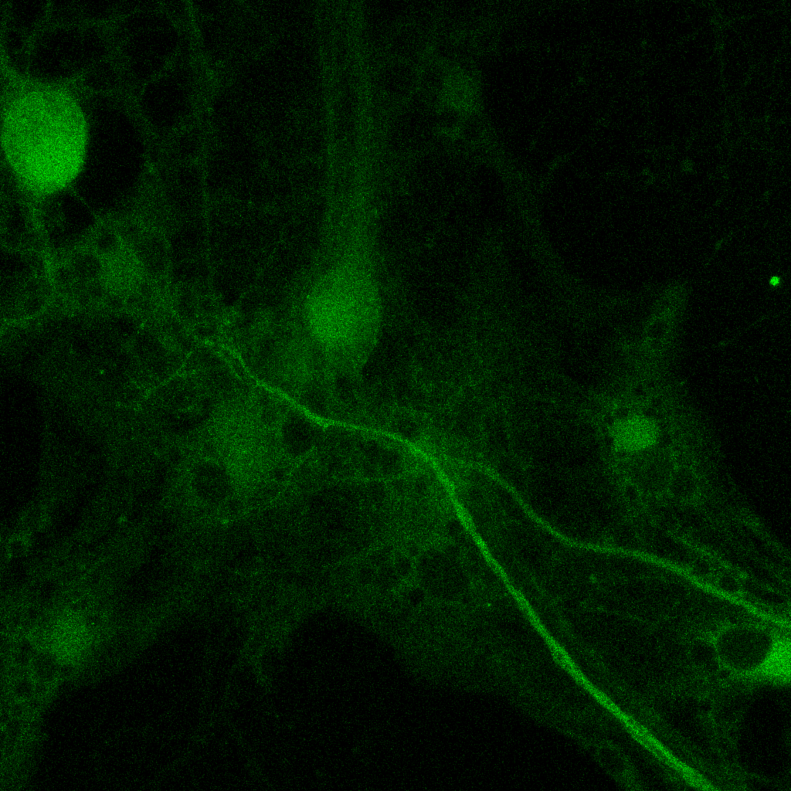

Supplement: Supplementary file 4 — Source data Fig. 3 [file 44318_2025_521_MOESM4_ESM.zip › Fig3/Fig3A/Images 3A/Fig.3 A Abeta.tif]

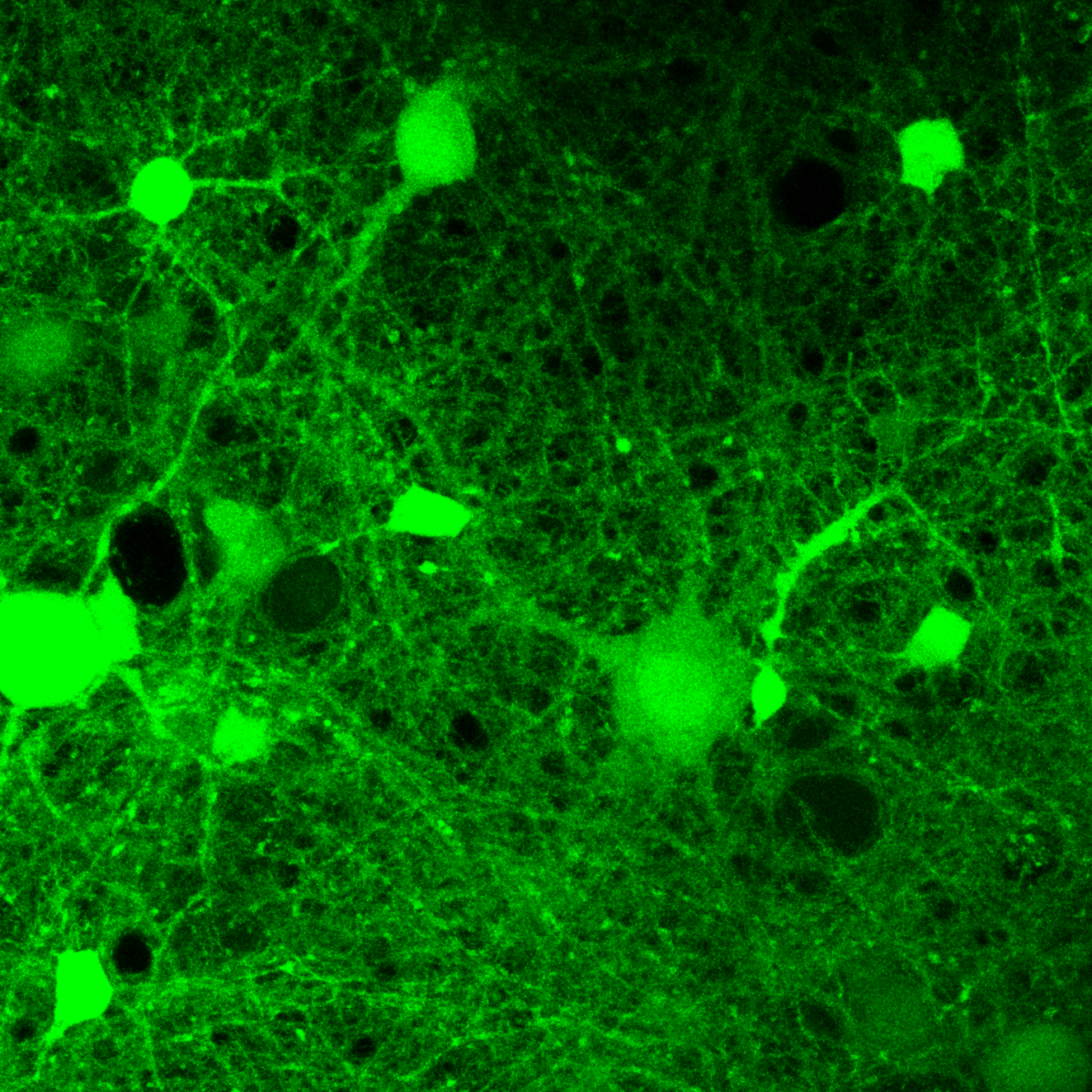

Supplement: Supplementary file 4 — Source data Fig. 3 [file 44318_2025_521_MOESM4_ESM.zip › Fig3/Fig3A/Images 3A/Fig.3 A H2O2.tif]

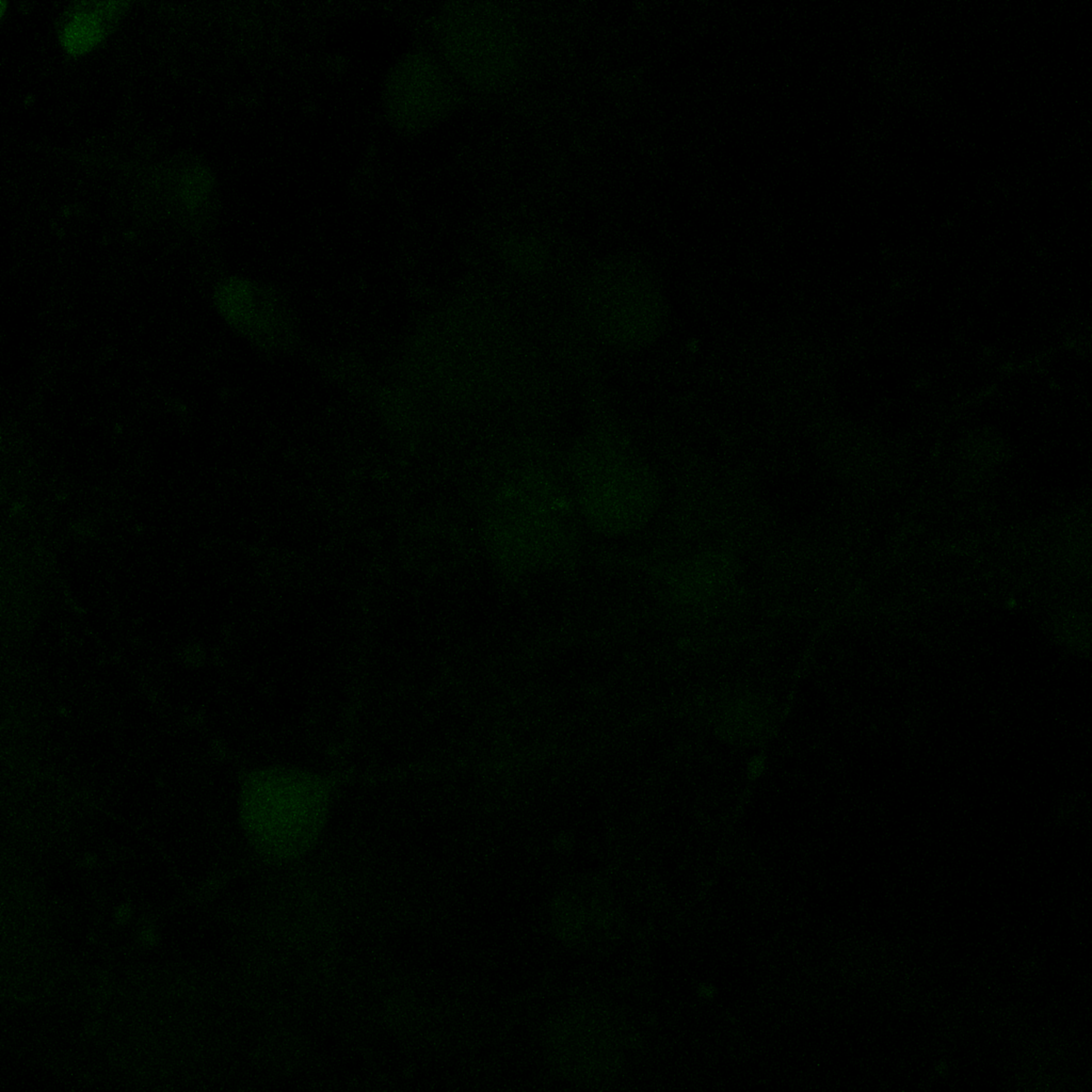

Supplement: Supplementary file 4 — Source data Fig. 3 [file 44318_2025_521_MOESM4_ESM.zip › Fig3/Fig3A/Images 3A/Fig.3 A Vehicle.tif]

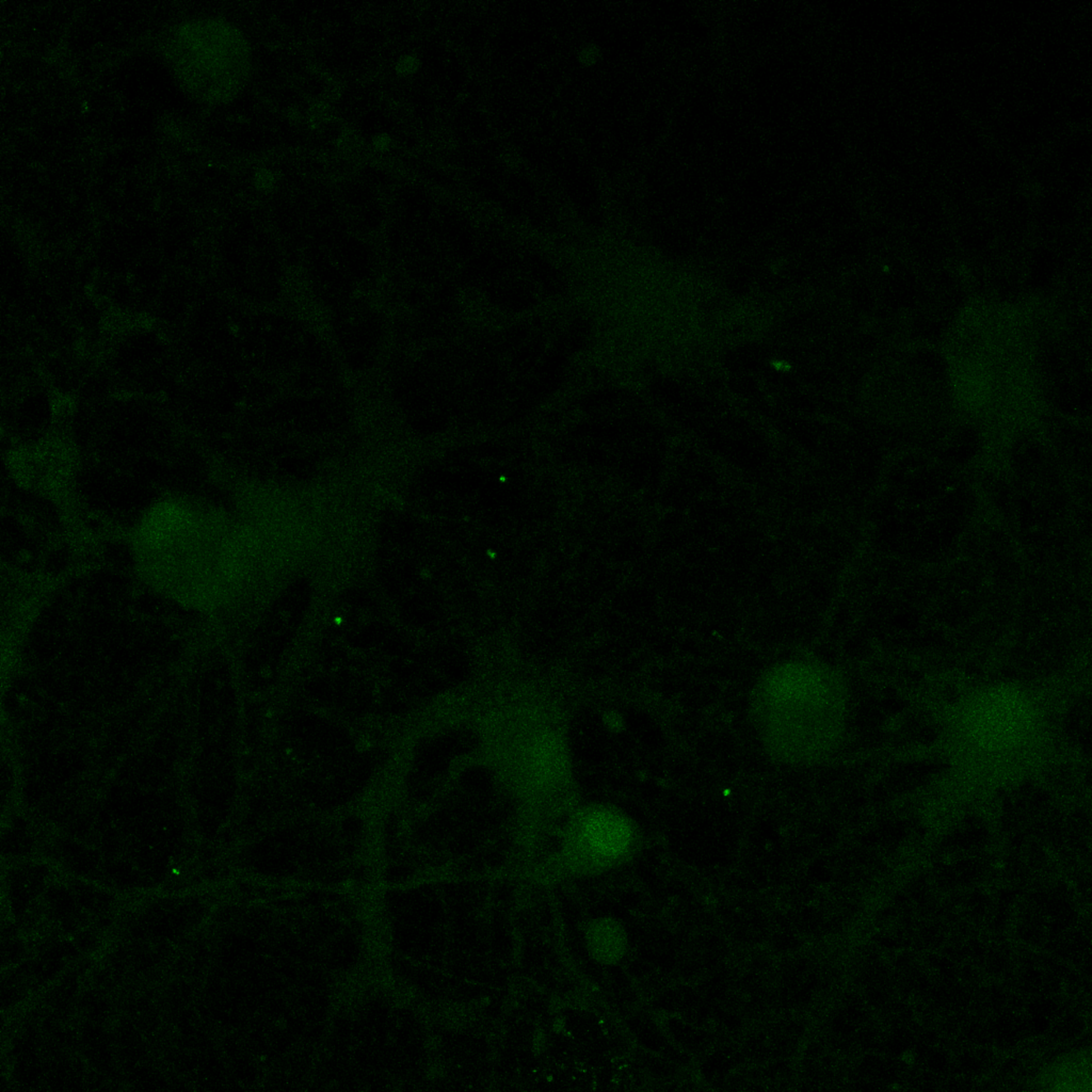

Supplement: Supplementary file 5 — Source data Fig. 4 [file 44318_2025_521_MOESM5_ESM.zip › Fig4/Fig4A/ImagesFig4A/Fig.4 A Abeta.tif]

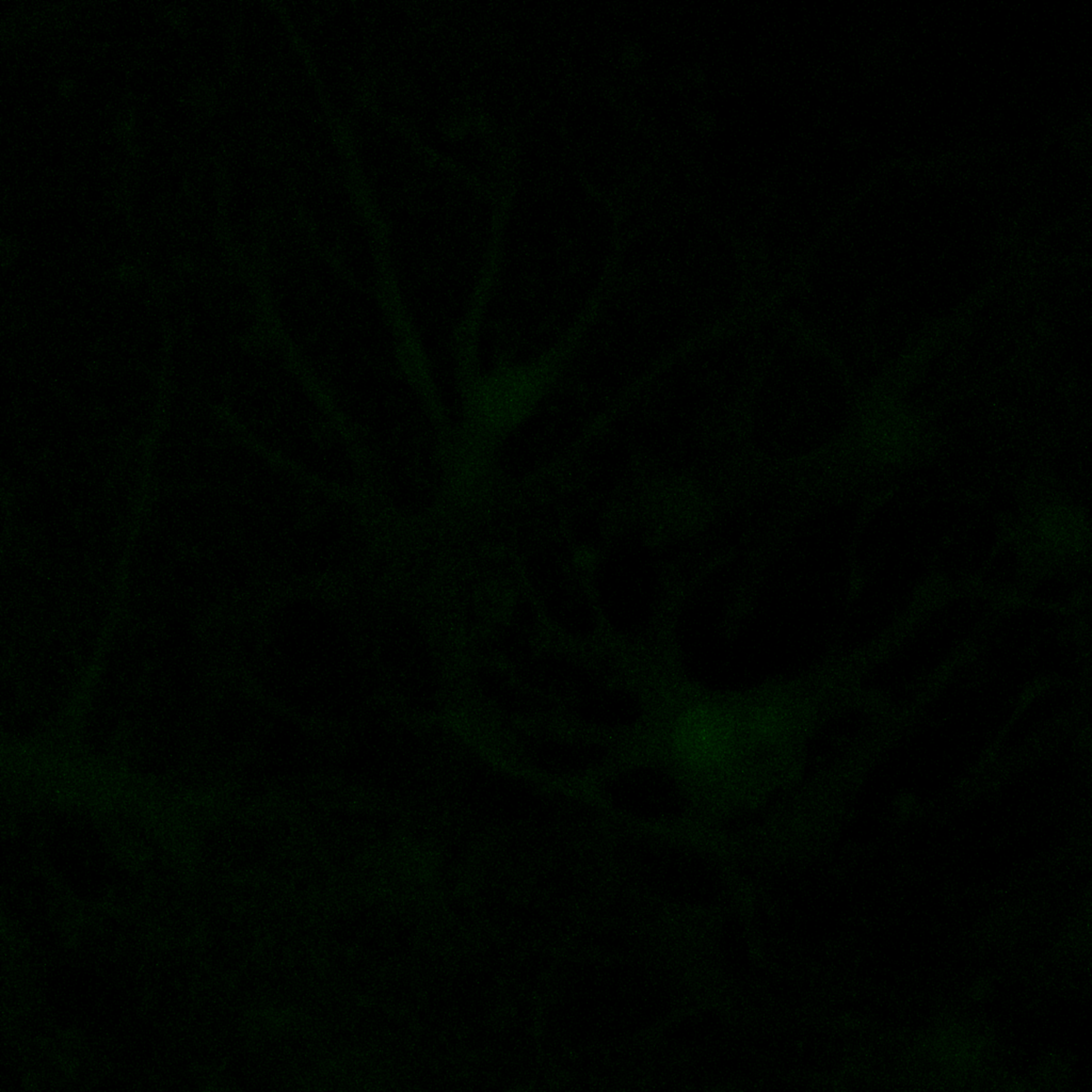

Supplement: Supplementary file 5 — Source data Fig. 4 [file 44318_2025_521_MOESM5_ESM.zip › Fig4/Fig4A/ImagesFig4A/Fig.4 A Abeta+EGTA.tif]

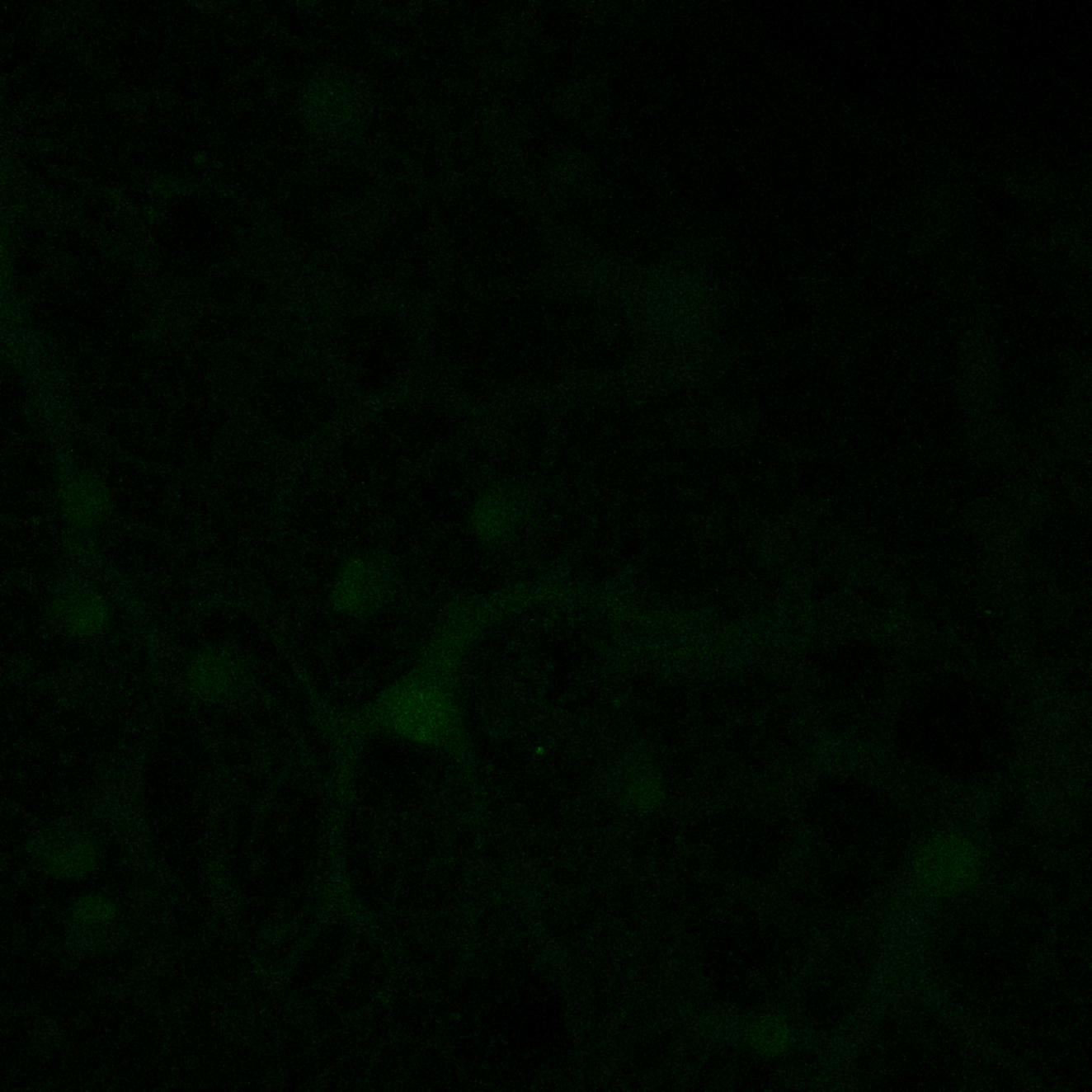

Supplement: Supplementary file 5 — Source data Fig. 4 [file 44318_2025_521_MOESM5_ESM.zip › Fig4/Fig4A/ImagesFig4A/Fig.4 A Abeta+Mem CNQX.tif]

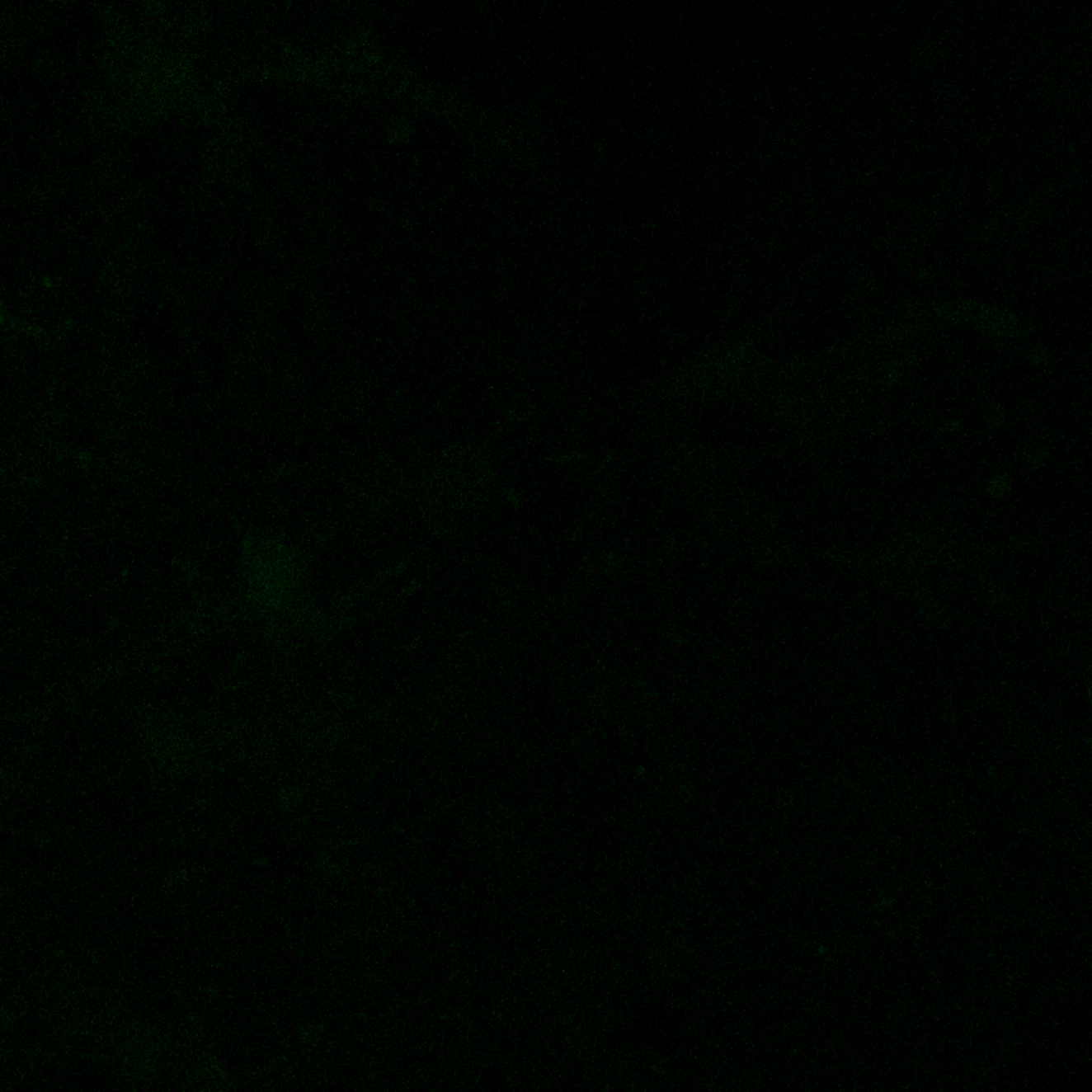

Supplement: Supplementary file 5 — Source data Fig. 4 [file 44318_2025_521_MOESM5_ESM.zip › Fig4/Fig4A/ImagesFig4A/Fig.4 A Vehicle.tif]

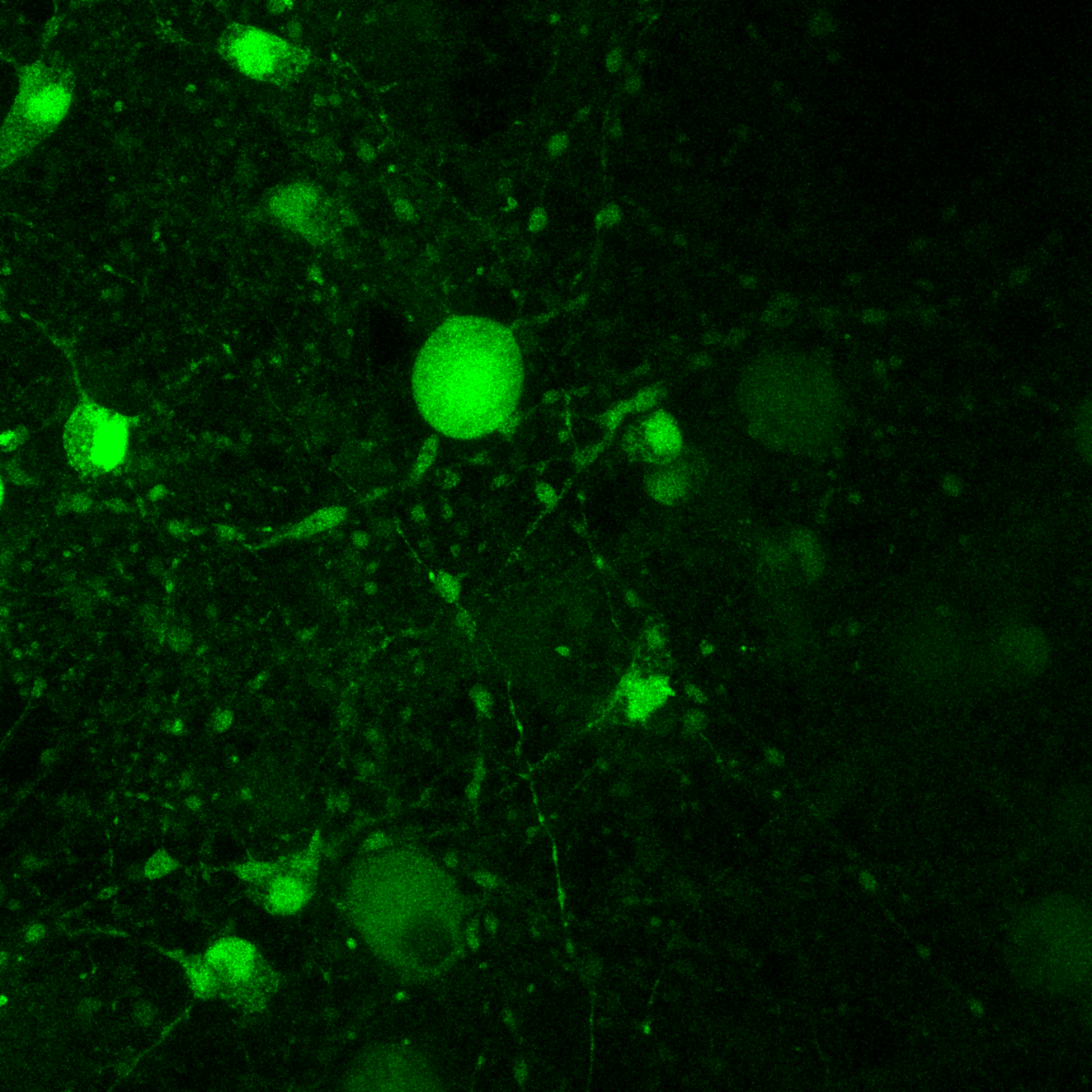

Supplement: Supplementary file 5 — Source data Fig. 4 [file 44318_2025_521_MOESM5_ESM.zip › Fig4/Fig4B/ImagesFig4B/Fig.4 B Abeta.tif]

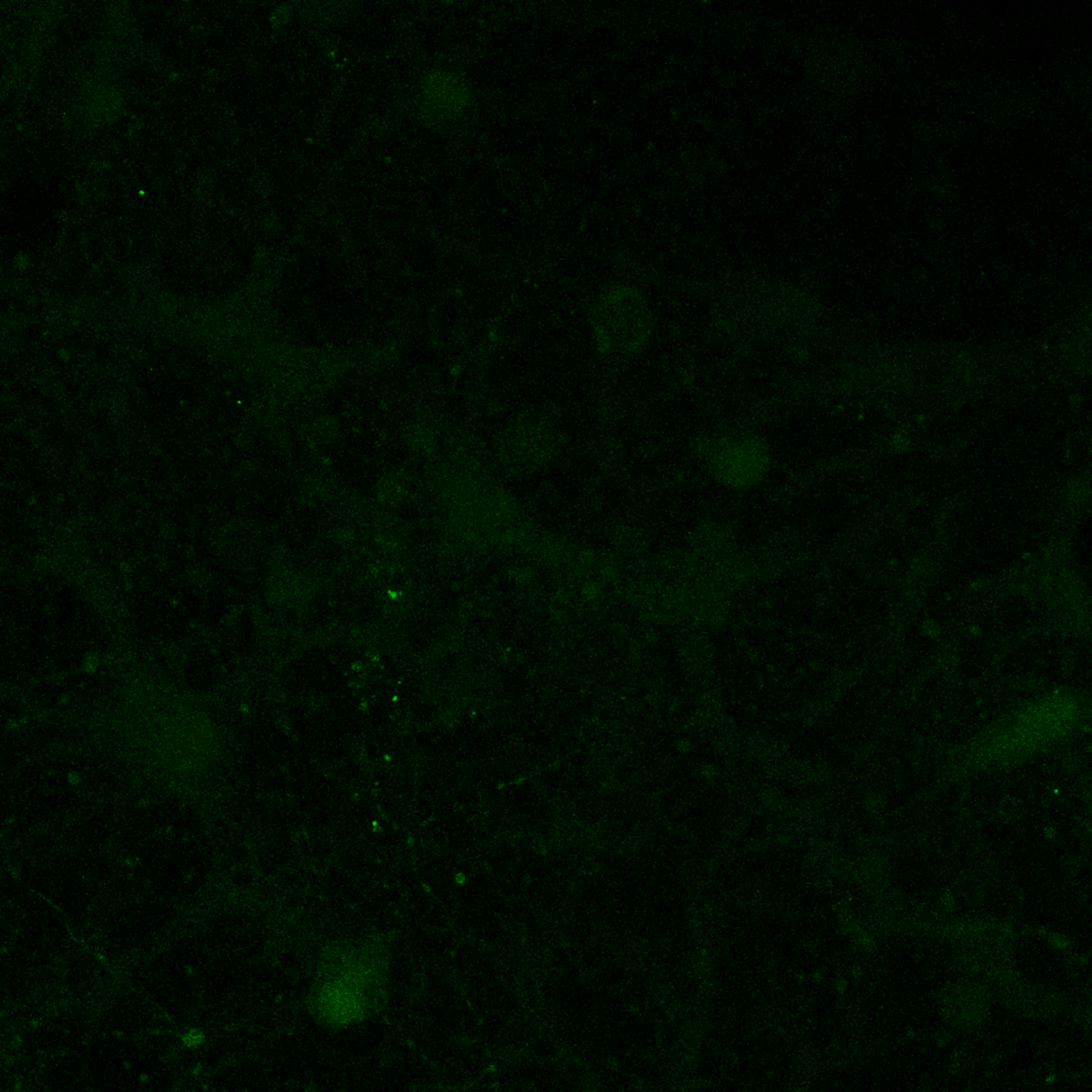

Supplement: Supplementary file 5 — Source data Fig. 4 [file 44318_2025_521_MOESM5_ESM.zip › Fig4/Fig4B/ImagesFig4B/Fig.4 B Abeta+EGTA.tif]

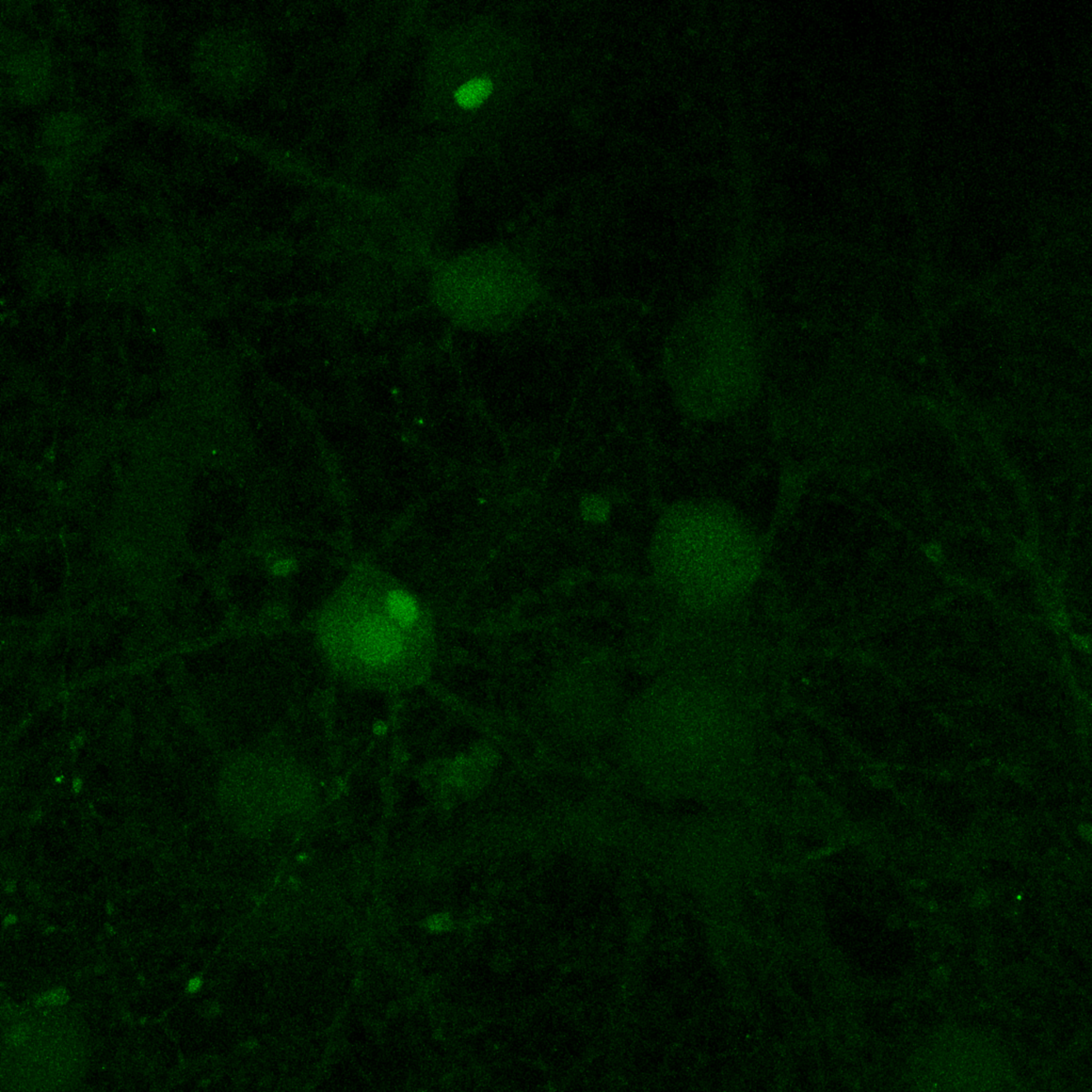

Supplement: Supplementary file 5 — Source data Fig. 4 [file 44318_2025_521_MOESM5_ESM.zip › Fig4/Fig4B/ImagesFig4B/Fig.4 B Abeta+Mem CNQX.tif]

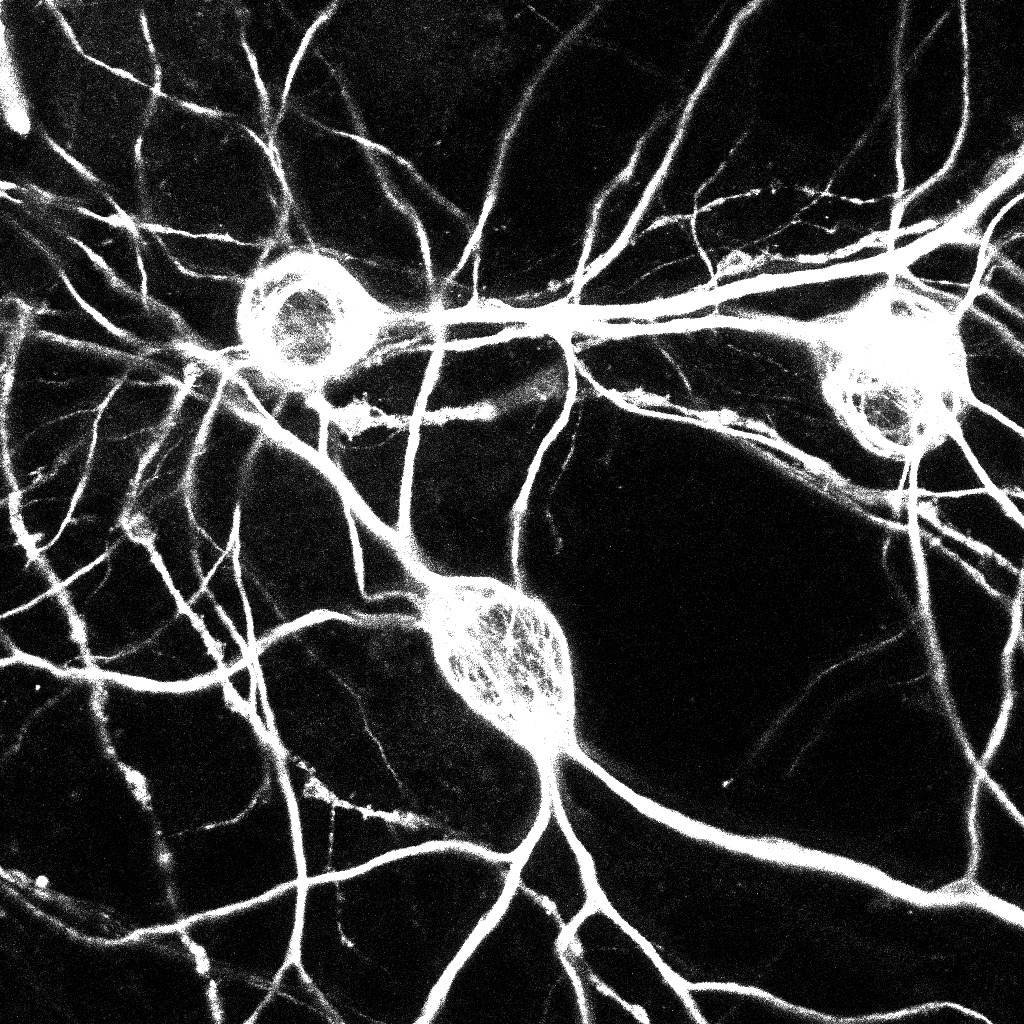

Supplement: Supplementary file 6 — Source data Fig. 5 [file 44318_2025_521_MOESM6_ESM.zip › Fig5/Fig5C/ImagesFig5C/ControlASO_MAP2.tif]

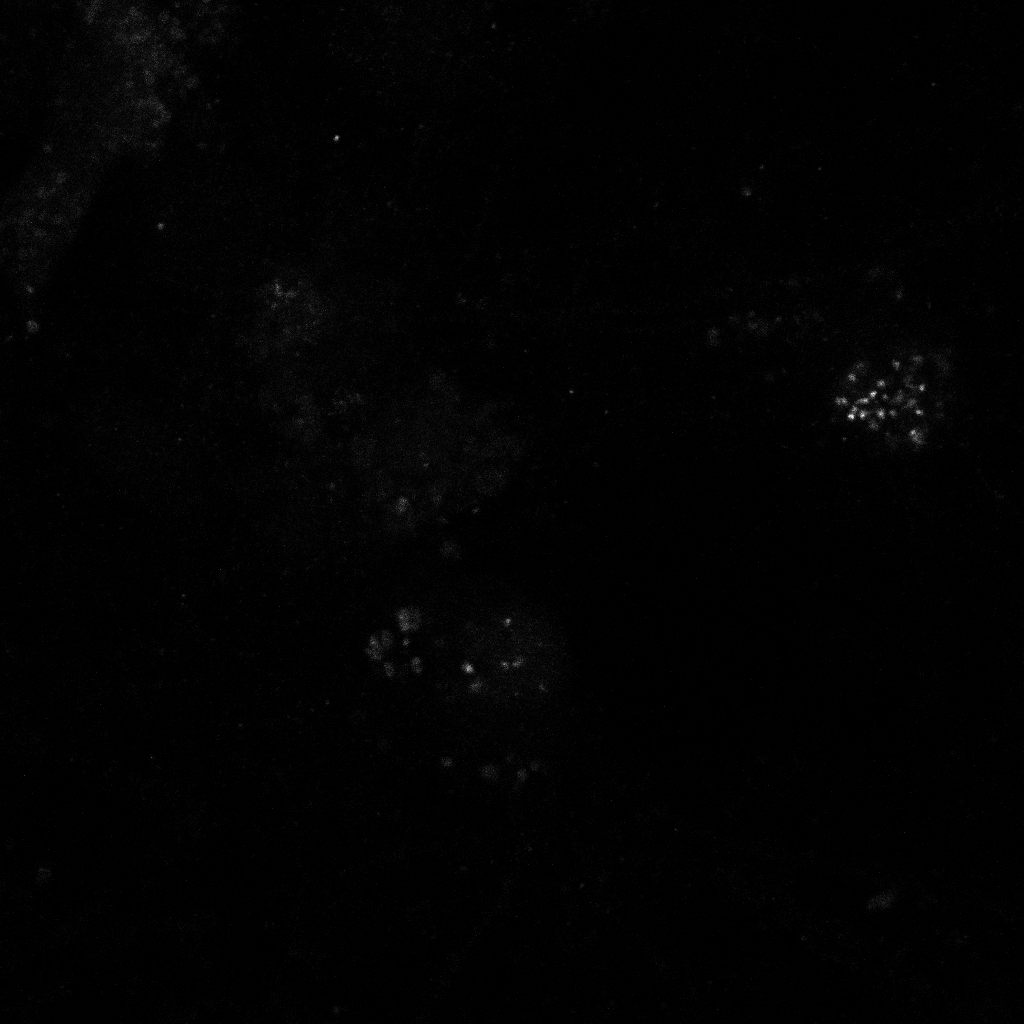

Supplement: Supplementary file 6 — Source data Fig. 5 [file 44318_2025_521_MOESM6_ESM.zip › Fig5/Fig5C/ImagesFig5C/ControlASO_gH2AX.tif]

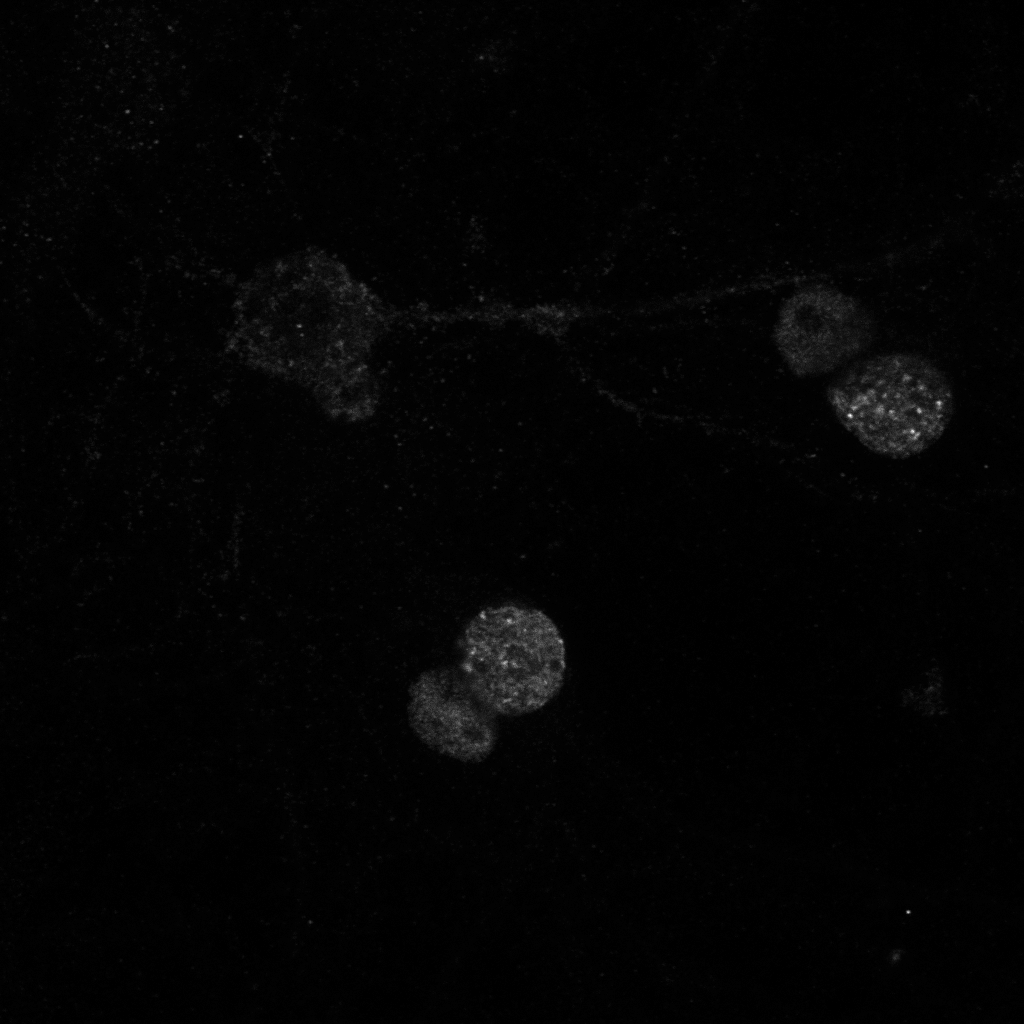

Supplement: Supplementary file 6 — Source data Fig. 5 [file 44318_2025_521_MOESM6_ESM.zip › Fig5/Fig5C/ImagesFig5C/ControlASO_53BP1.tif]

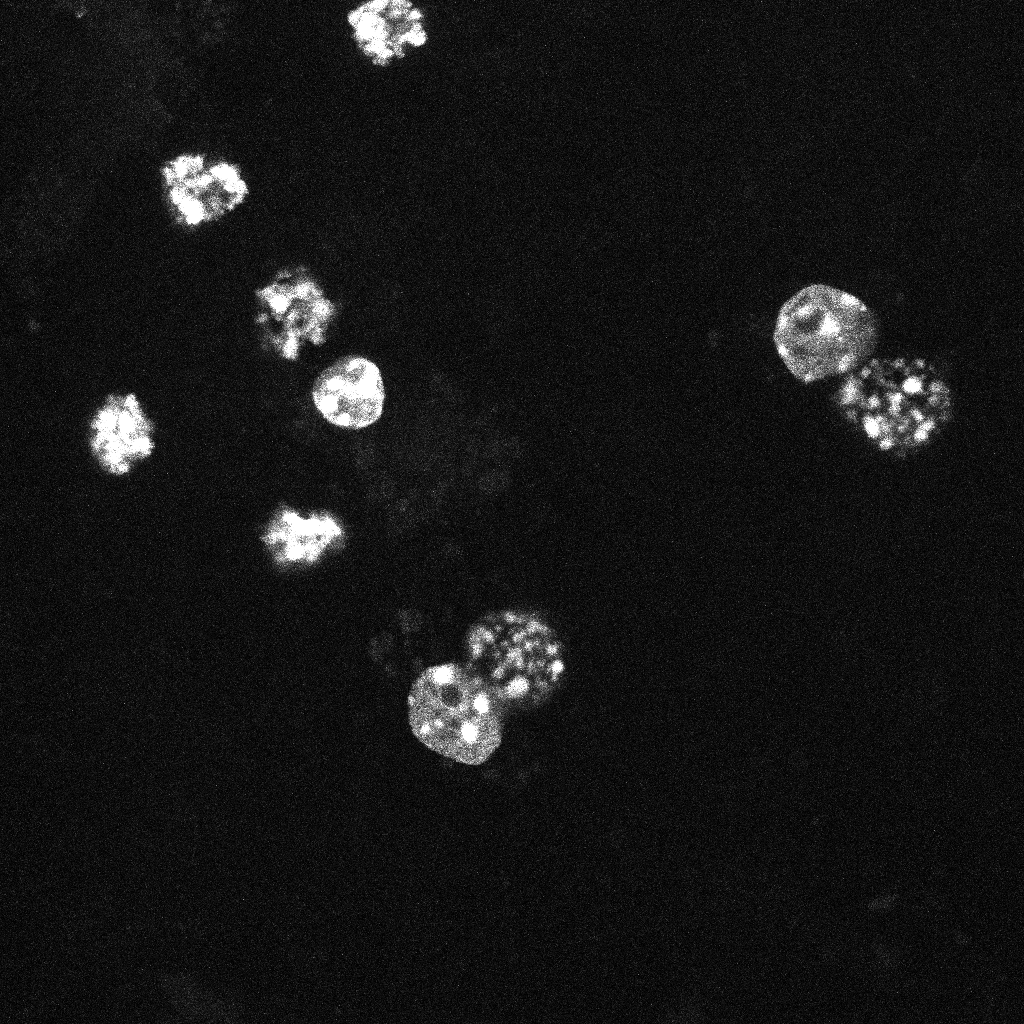

Supplement: Supplementary file 6 — Source data Fig. 5 [file 44318_2025_521_MOESM6_ESM.zip › Fig5/Fig5C/ImagesFig5C/ControlASO_DAPI.tif]

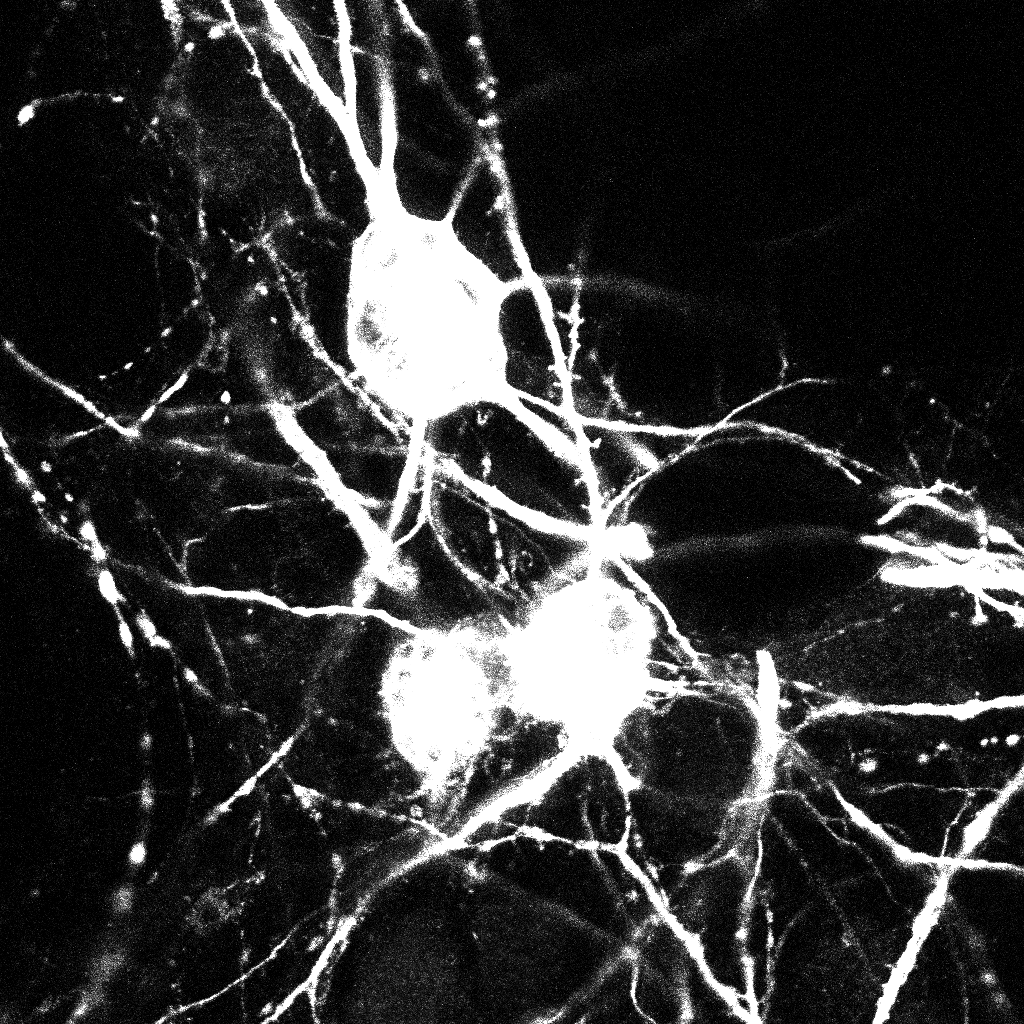

Supplement: Supplementary file 6 — Source data Fig. 5 [file 44318_2025_521_MOESM6_ESM.zip › Fig5/Fig5C/ImagesFig5C/ASO_Anti-TeloG_MAP2.tif]

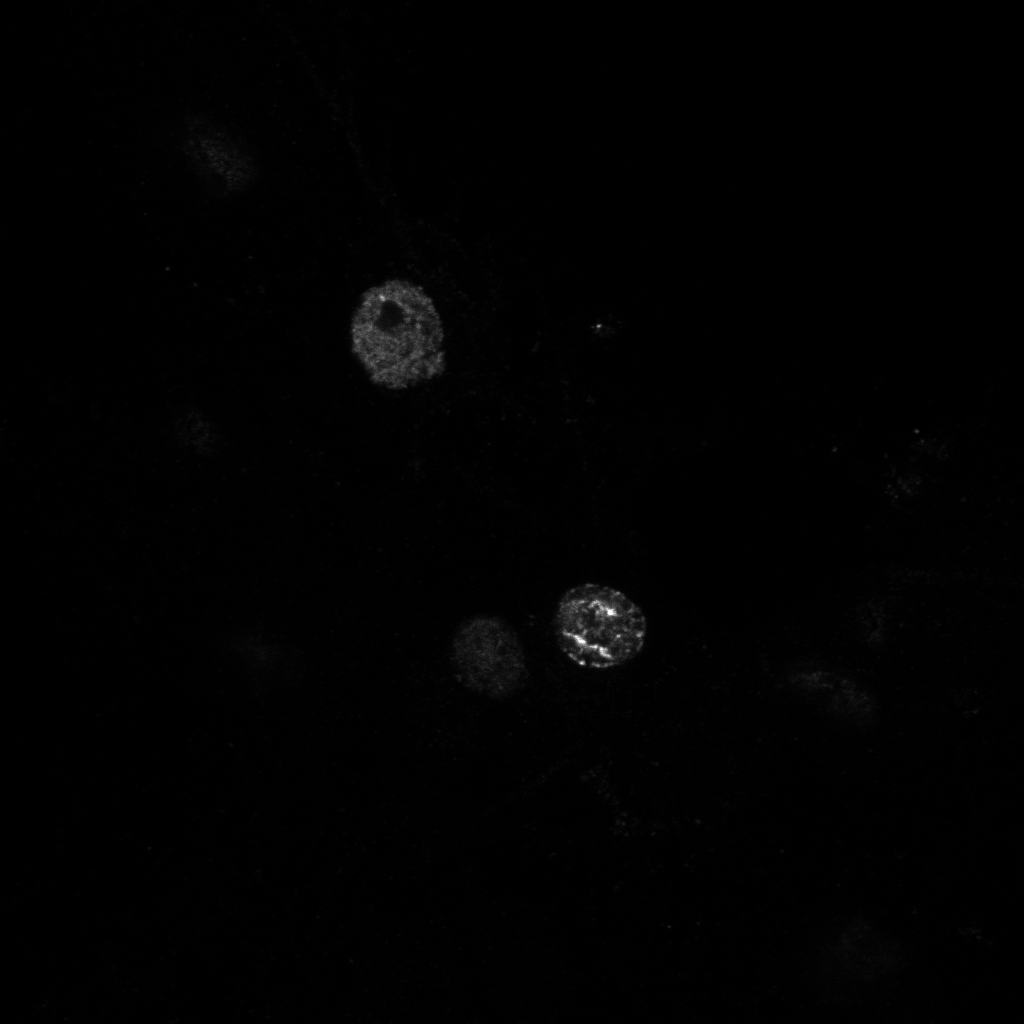

Supplement: Supplementary file 6 — Source data Fig. 5 [file 44318_2025_521_MOESM6_ESM.zip › Fig5/Fig5C/ImagesFig5C/ASO_Anti-TeloG_53BP1.tif]

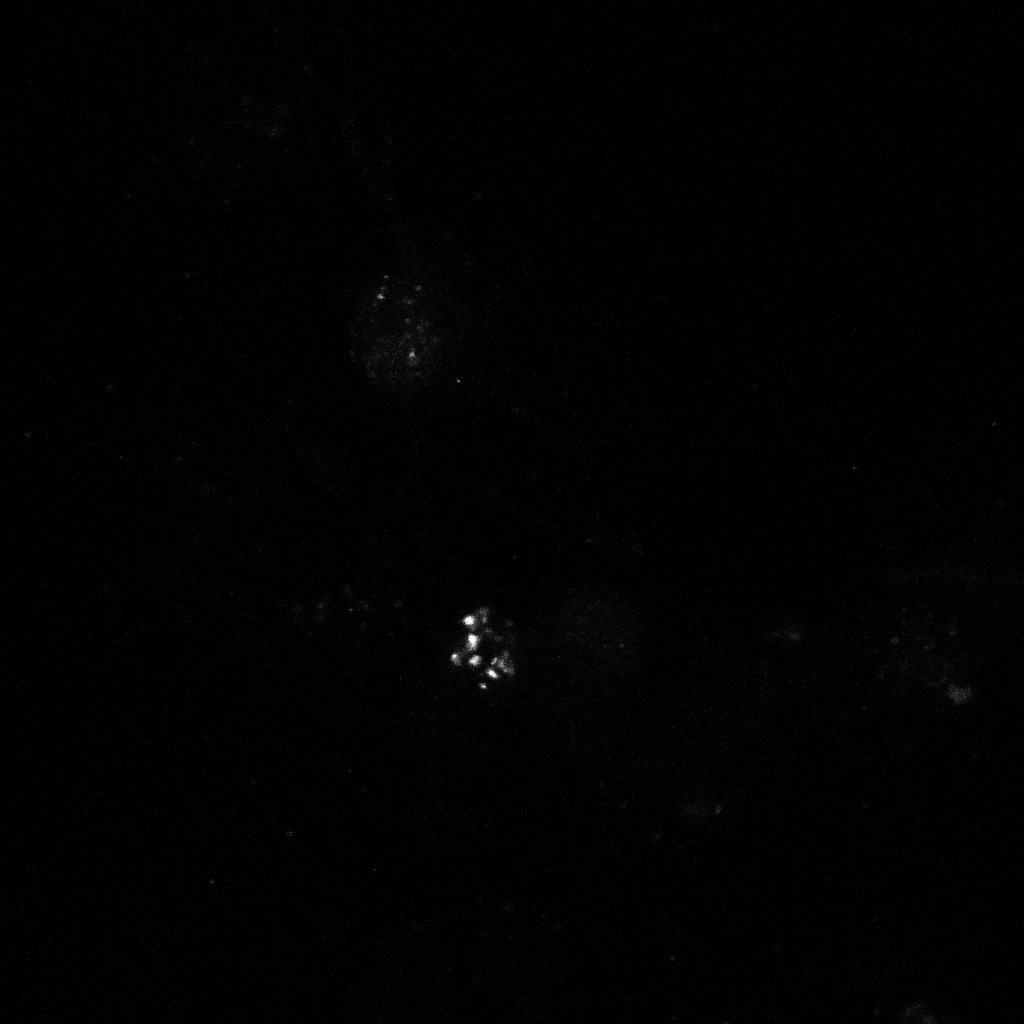

Supplement: Supplementary file 6 — Source data Fig. 5 [file 44318_2025_521_MOESM6_ESM.zip › Fig5/Fig5C/ImagesFig5C/ASO_Anti-TeloG_gH2AX.tif]

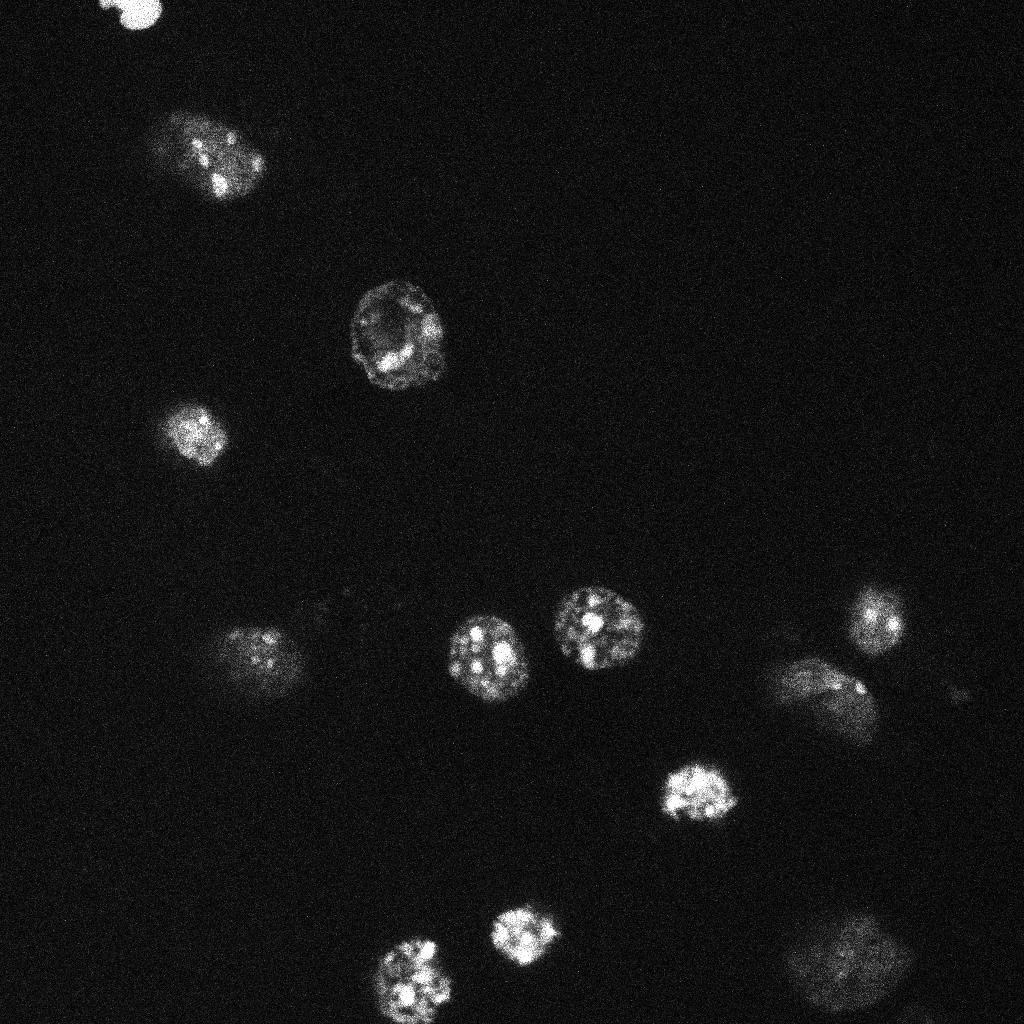

Supplement: Supplementary file 6 — Source data Fig. 5 [file 44318_2025_521_MOESM6_ESM.zip › Fig5/Fig5C/ImagesFig5C/ASO_Anti-TeloG_DAPI.tif]

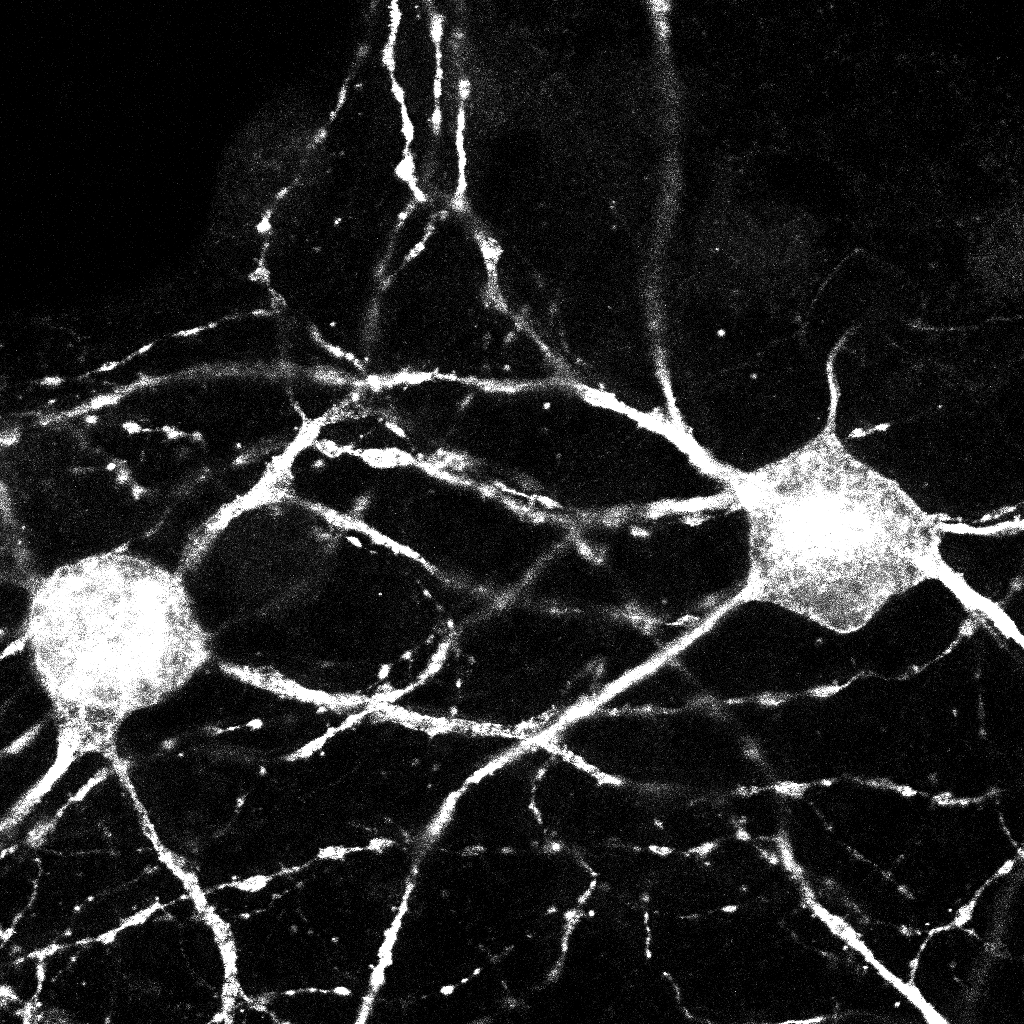

Supplement: Supplementary file 6 — Source data Fig. 5 [file 44318_2025_521_MOESM6_ESM.zip › Fig5/Fig5C/ImagesFig5C/ASO_Anti-TeloC_MAP2.tif]

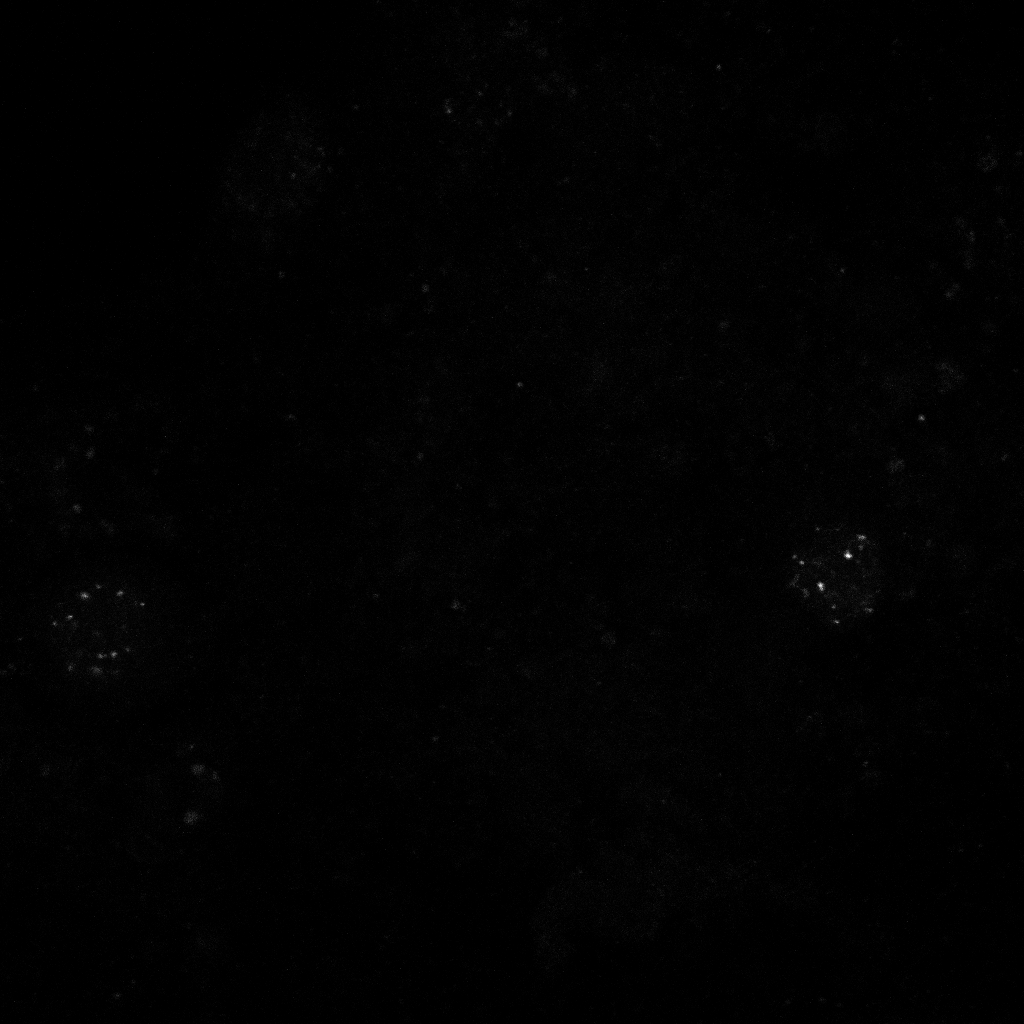

Supplement: Supplementary file 6 — Source data Fig. 5 [file 44318_2025_521_MOESM6_ESM.zip › Fig5/Fig5C/ImagesFig5C/ASO_Anti-TeloC_gH2AX.tif]

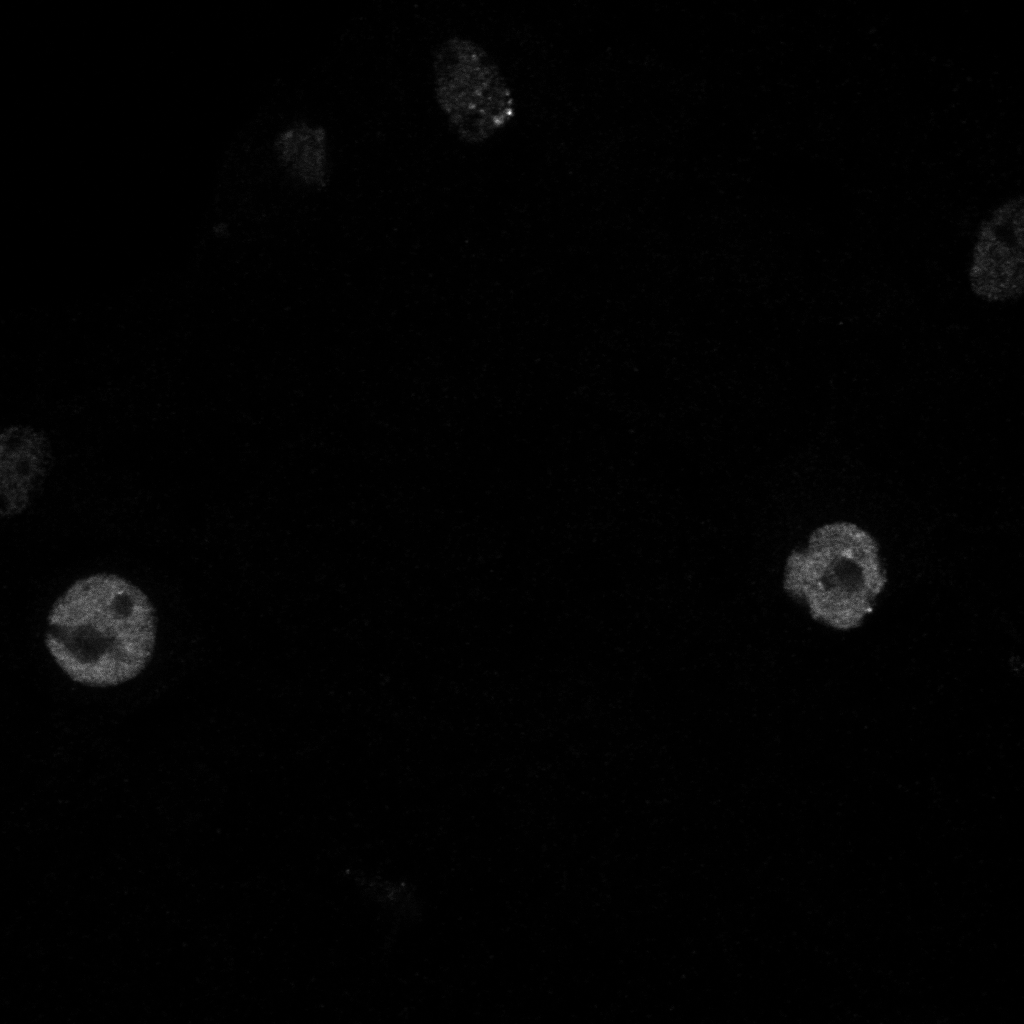

Supplement: Supplementary file 6 — Source data Fig. 5 [file 44318_2025_521_MOESM6_ESM.zip › Fig5/Fig5C/ImagesFig5C/ASO_Anti-TeloC_53BP1.tif]

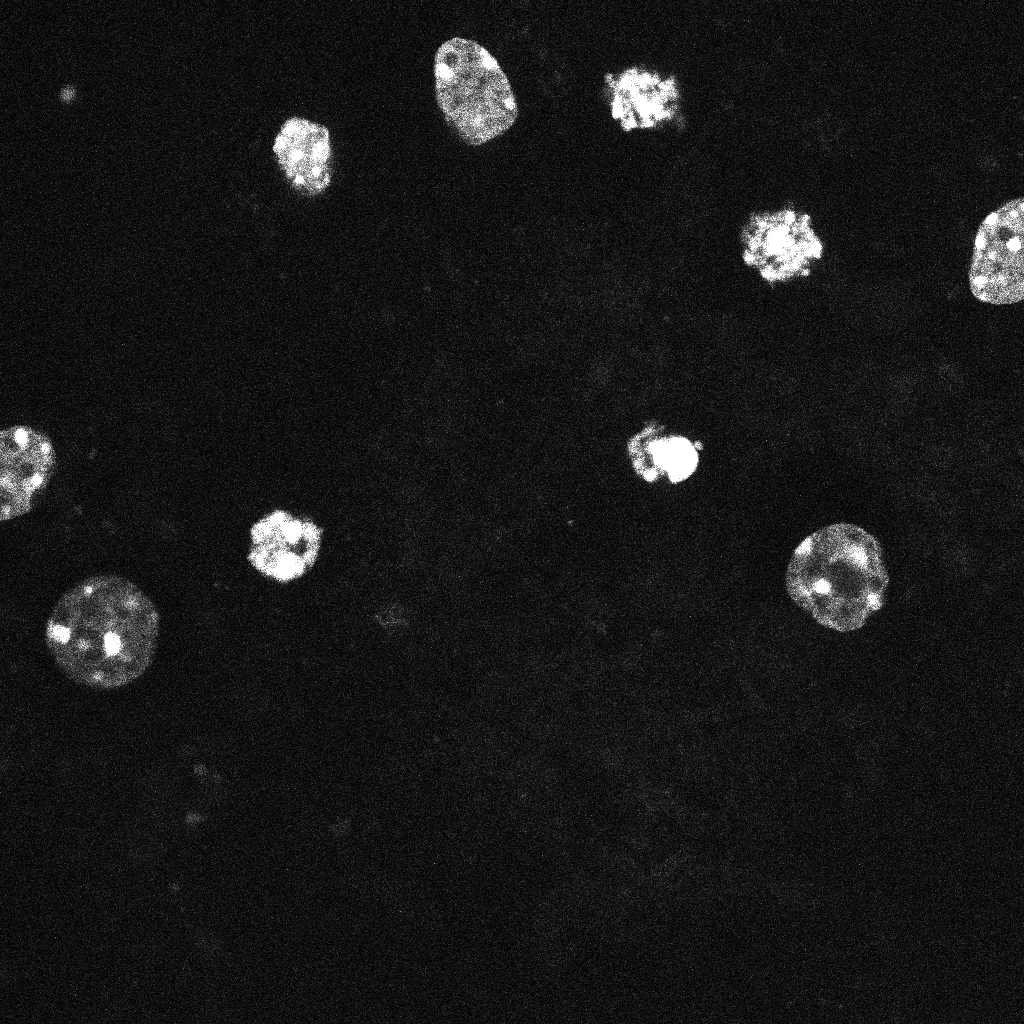

Supplement: Supplementary file 6 — Source data Fig. 5 [file 44318_2025_521_MOESM6_ESM.zip › Fig5/Fig5C/ImagesFig5C/ASO_Anti-TeloC_DAPI.tif]

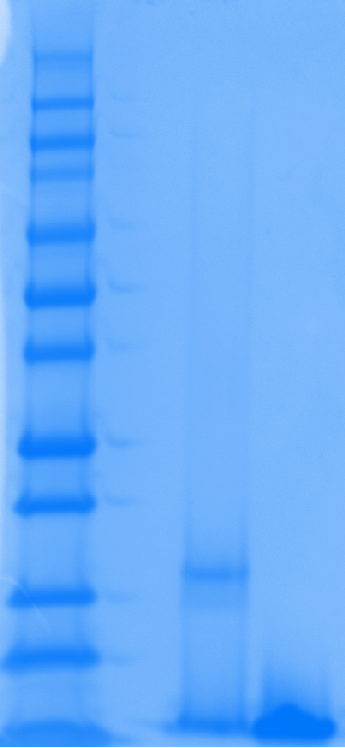

Supplement: Supplementary file 9 — Figure EV2B Source Data [file 44318_2025_521_MOESM9_ESM.zip › Images Fig EV2B/Blue comassei gel.tif]
