# Supplementary material for: Telomeric DNA damage response mediates neurotoxicity of Aβ42 oligomers in Alzheimer’s disease
Source: EMBO J. 2025 Sep 21;44(21):6078–111. doi: 10.1038/s44318-025-00521-1 (PMC12583505; doi:10.1038/s44318-025-00521-1)
Supplement: Supplementary file 9 — Figure EV2B Source Data [file 44318_2025_521_MOESM9_ESM.zip › Images Fig EV2B/Western blot Abeta oligomers .pptx]

## Slide 1
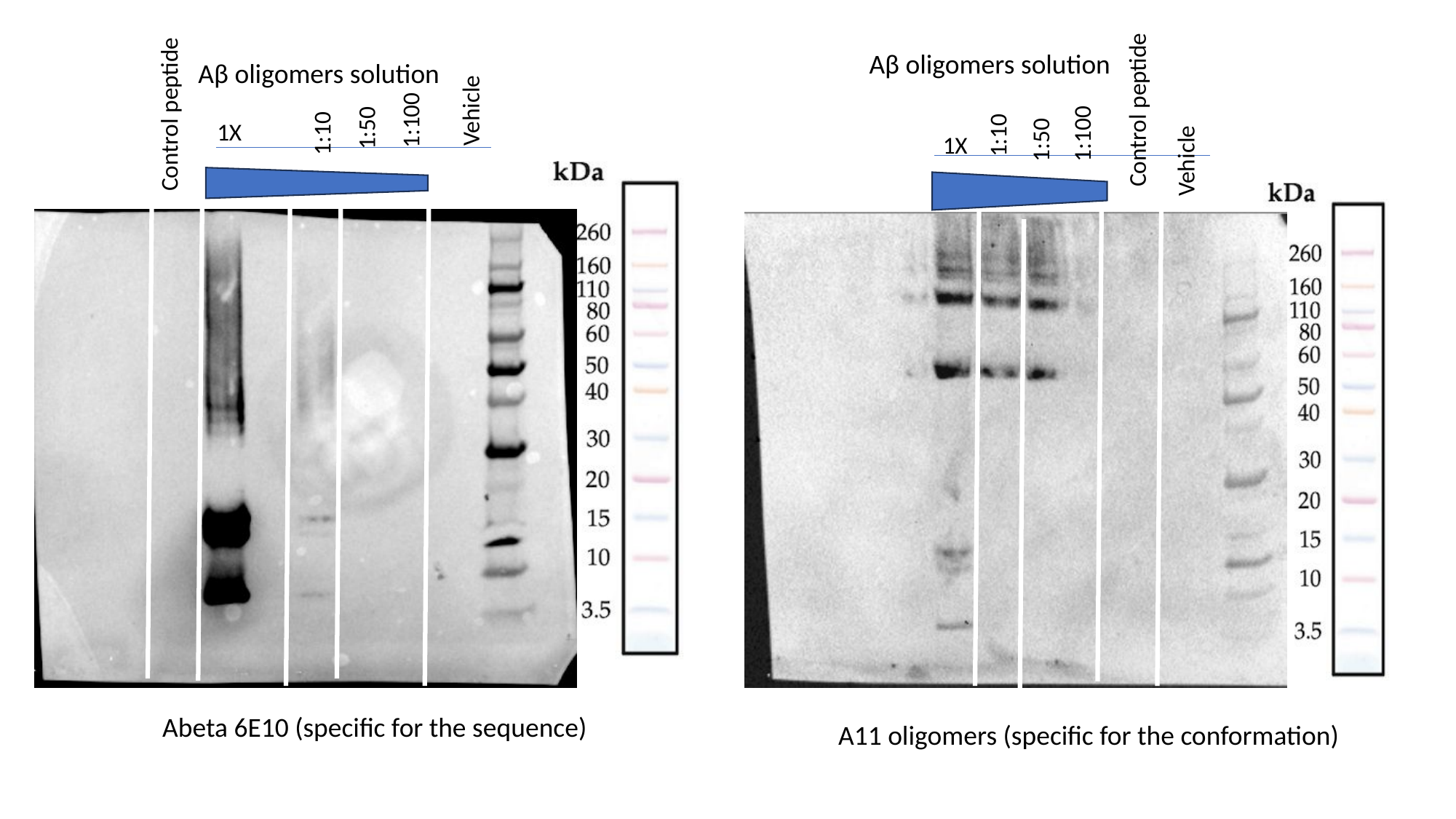

Aβ oligomers solution
Aβ oligomers solution
Vehicle
Control peptide
Control peptide
1:100
1:50
1:10
1:10
1:100
1:50
1X
1X
Vehicle
Abeta 6E10 (specific for the sequence)
A11 oligomers (specific for the conformation)
