# Supplementary material for: Telomeric DNA damage response mediates neurotoxicity of Aβ42 oligomers in Alzheimer’s disease
Source: EMBO J. 2025 Sep 21;44(21):6078–111. doi: 10.1038/s44318-025-00521-1 (PMC12583505; doi:10.1038/s44318-025-00521-1)
Supplement: Supplementary file 10 — Expanded View Figures [file 44318_2025_521_MOESM10_ESM.pdf]

Expanded View Figures

Figure EV1. Markers of tDDR activation and pro-apoptotic gene expression are elevated in the hippocampus of 3xTg-AD mice.

(**A–D**) Hippocampal regions from 12-month-old wt and 3xTg-AD mice were analyzed. Each circle in the graphs represents one mouse analyzed. (**A**) Representative confocal images of immunofluorescence staining of hippocampal regions from wt and 3xTg-AD mice with antibodies against γH2AX, 53BP1, MAP2, nuclei were stained with DAPI. Bar graph shows quantification of the average number of γH2AX ($n = 4$ wt and $n = 4$ 3xTg-AD), and 53BP1 foci per nucleus ($n = 5$ wt and $n =$ 53xTg-AD). At least 50 nuclei per mouse section were scored. (**B**) Bar graphs show the quantification of G-rich ($n = 4$ wt and $n = 8$ 3xTg-AD) and C-rich ($n = 5$ wt and $n = 10$ 3xTg-AD) tdincRNA levels in 3xTg-AD mice relative to wt samples in brain hippocampus as measured by RT-qPCR. (**C**) Bar graph shows the quantification of p21 mRNA levels in 3xTg-AD mice relative to wt samples in the hippocampus as measured by RT-qPCR. ($n = 4$ wt and $n = 4$ 3xTg-AD). (**D**) Bar graph represents the quantification of the ratio between the mRNA levels of Bax over Bcl-2 in 3xTg-AD mice relative to wt samples in the hippocampus as measured by RT-qPCR ($n = 4$ wt and $n = 4$ 3xTg-AD). (**E**) Representative confocal microscopy images of Immunofluorescence stainings combining antibodies against MAP2, BAX and BCL-2 of sections from wt and 3xTg-AD mice (left panel). Bar graph represents the quantification of the ratio between the signal of BAX over BCL-2 from immunofluorescence in 3xTg-AD mice relative to wt samples in hippocampal regions ($n = 4$ wt and $n = 4$ 3xTg-AD). Unpaired *t* test was applied and data are represented as mean ± SEM.

© The Author(s)

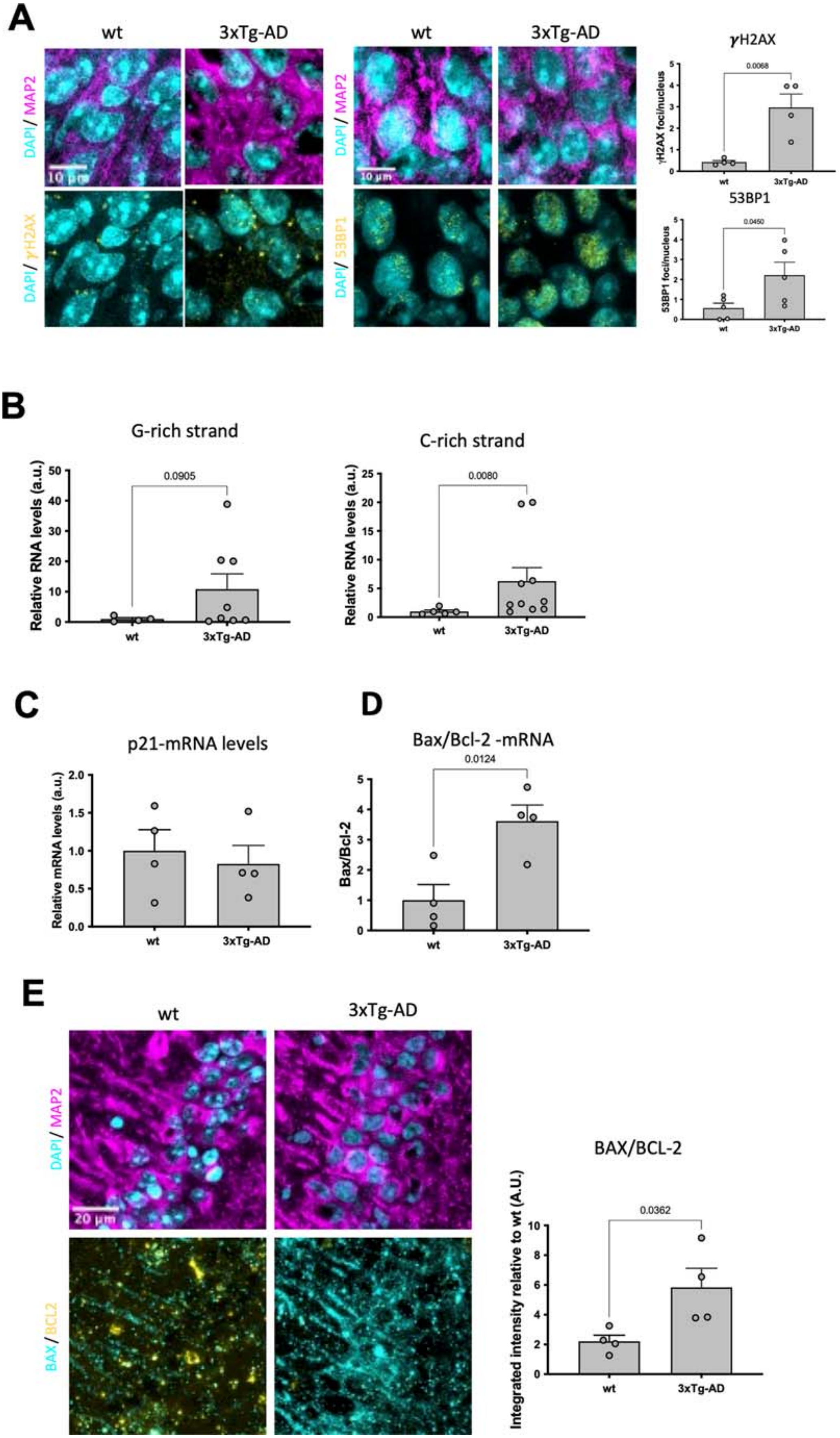
A
wt
3xTg-AD
DAPI/ MAP2
DAPI/ γH2AX
DAPI/ 53BP1
10 μm
γH2AX
0.0068
γH2AX foci/nucleus
53BP1
0.0450
53BP1 foci/nucleus
wt
3xTg-AD
B
G-rich strand
C-rich strand
0.0905
0.0080
Relative RNA levels (a.u.)
C
p21-mRNA levels
Relative mRNA levels (a.u.)
D
Bax/Bcl-2 -mRNA
0.0124
Bax/Bcl-2
E
DAPI/ MAP2
BAX/ BCL2
20 μm
BAX/BCL-2
0.0362
Integrated intensity relative to wt (A.U.)

 © The Author(s)

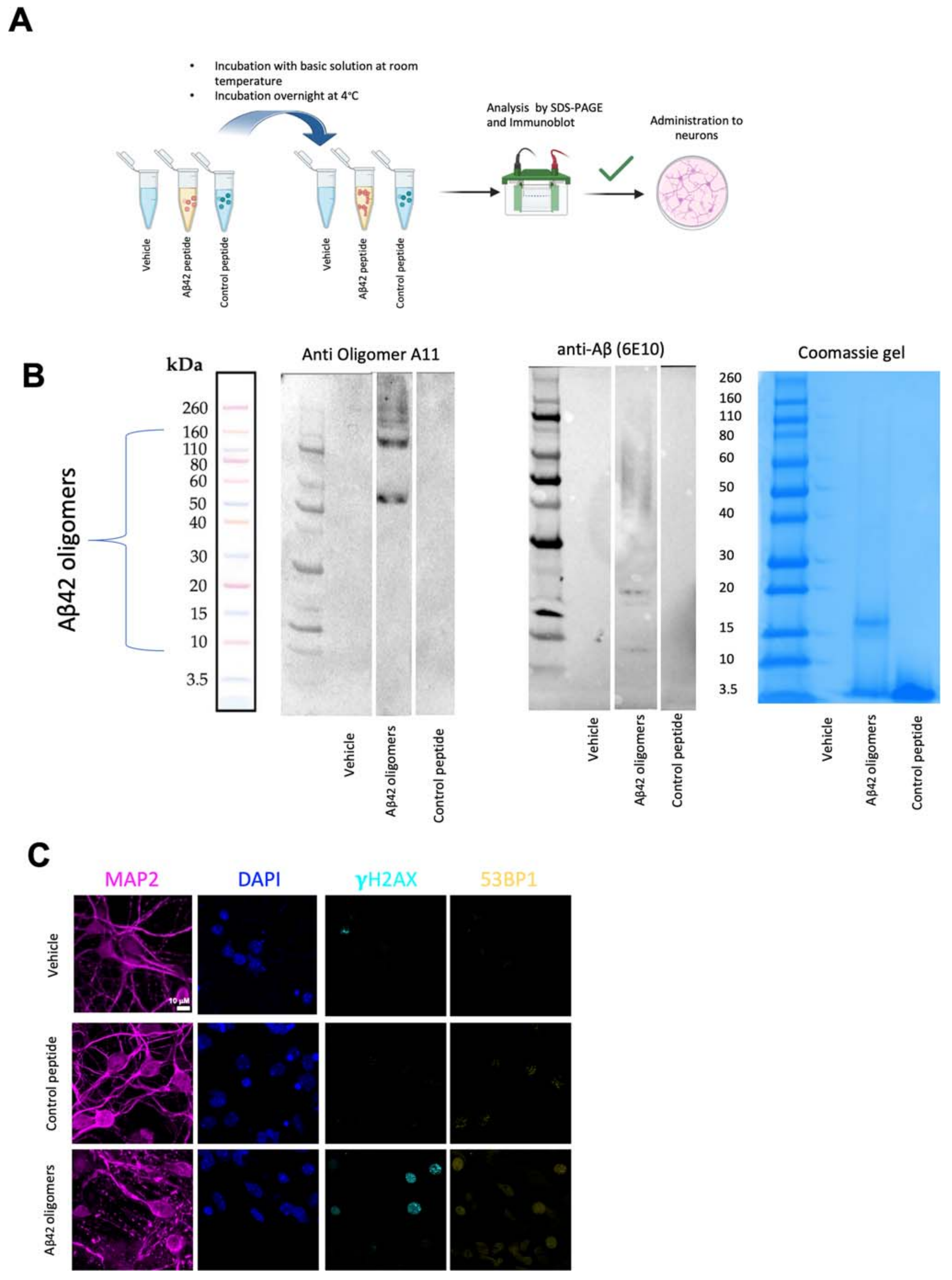
A
Incubation with basic solution at room temperature
Incubation overnight at 4°C
Analysis by SDS-PAGE and Immunoblot
Administration to neurons
Vehicle
Aβ42 peptide
Control peptide
B
kDa
Aβ42 oligomers
260
160
110
80
60
50
40
30
20
15
10
3.5
Anti Oligomer A11
anti-Aβ (6E10)
Coomassie gel
Vehicle
Aβ42 oligomers
Control peptide
C
MAP2
DAPI
γH2AX
53BP1
Vehicle
Control peptide
Aβ42 oligomers
10 μM

Figure EV2. Experimental descriptions of Aβ42 oligomers preparation and their specificity.

In all experiments in this manuscript, a fresh batch of Aβ42 oligomers was prepared as described in the "Methods and protocols" section. (**A**) Scheme of Aβ42 oligomer preparation and analysis of the oligomer's solution. Each Aβ42 oligomers batch was tested by immunoblotting for oligomerization status before use. (**B**) Representative images of western blots using the Oligomers A11 polyclonal antibody recognizing selectively the oligomeric forms only, or the 6E10 monoclonal antibody recognizing the C terminal fragment (CTF) of the peptide. Control peptide prepared in parallel but unable to undergo oligomerization, was used as negative control. Polyacrylamide gel stained with blue Coomassie-based solution was used to check the Aβ42 oligomers and control peptide. (**C**) Representative images of immunofluorescence staining of mouse primary neurons treated with Vehicle, Aβ42 oligomers and solutions containing control peptide and stained with antibodies against MAP2 (magenta) as neuronal marker, γH2AX (cyan) and 53BP1 (yellow) as DDR markers, nuclei were stained with DAPI. Source data are available online for this figure.

 © The Author(s)

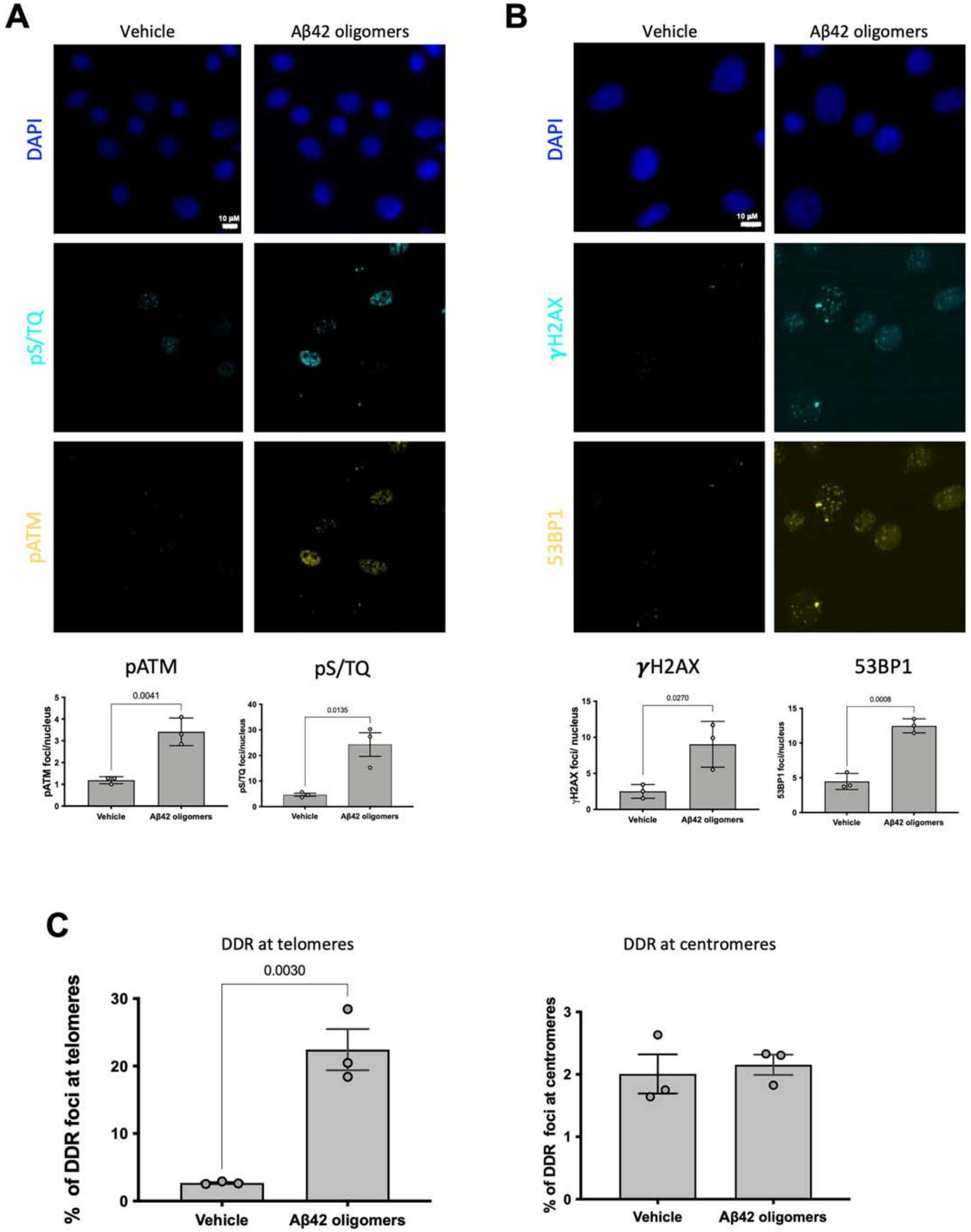
A
Vehicle
Aβ42 oligomers
DAPI
pS/TQ
pATM
10 μM
B
Vehicle
Aβ42 oligomers
DAPI
γH2AX
53BP1
pATM
pS/TQ
γH2AX
53BP1
pATM foci/nucleus
0.0041
pS/TQ foci/nucleus
0.0135
γH2AX foci/ nucleus
0.0270
53BP1 foci/nucleus
0.0008
Vehicle
Aβ42 oligomers
C
DDR at telomeres
0.0030
% of DDR foci at telomeres
DDR at centromeres
% of DDR foci at centromeres
Vehicle
Aβ42 oligomers

Figure EV3. Aβ42 oligomers induce DDR and accumulation at telomeres in HT-22 cells.

(**A–C**) HT-22 cells were treated with 5 μM Aβ42 oligomers for 24 h or vehicle. Each circle represents an independent biological replicate. (**A**) Representative confocal image of immunofluorescence staining of HT-22 with antibodies against pS/TQ (cyan) and pATM (yellow) as DDR markers, nuclei were stained with DAPI. Bar graphs show the quantification of average number of DDR foci per nucleus. At least 50 cells for each condition in each replicate were analyzed. ($n = 3$ for each condition) (**B**) Representative confocal images of immunofluorescence staining of HT-22 with antibodies against γH2AX (cyan) and 53BP1 (yellow) as DDR markers, nuclei were stained with DAPI. Bar graphs show the average number of DDR foci per nucleus. At least 50 cells for each condition in each replicate were analyzed. ($n = 3$ for each condition). (**C**) Bar graphs show the percentage of DDR foci that localize at telomeres or centromeres, as detected by immunoFISH combining γH2AX antibody and FISH for telomeric DNA using a complementary PNA probe and by double immunofluorescence combining antibodies against γH2AX and the centromeric protein CREST of primary neurons. At least 50 cells for each condition in each replicate were analyzed. ($n = 3$ for each condition). Unpaired *t* test was applied and data are represented as mean ± SEM.

© The Author(s)

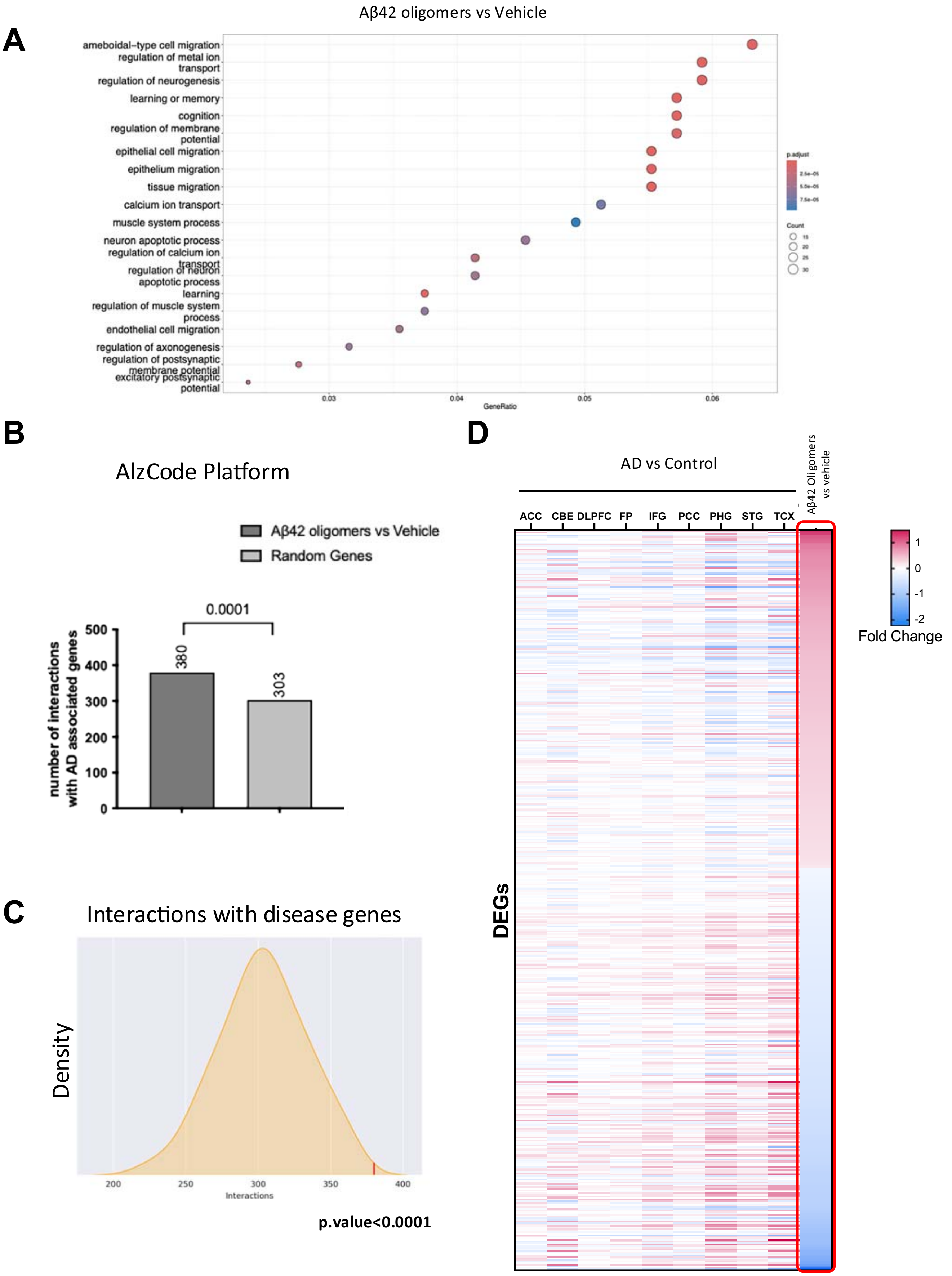
A
Aβ42 oligomers vs Vehicle
ameboidal-type cell migration
regulation of metal ion transport
regulation of neurogenesis
learning or memory
cognition
regulation of membrane potential
epithelial cell migration
epithelium migration
tissue migration
calcium ion transport
muscle system process
neuron apoptotic process
regulation of calcium ion transport
regulation of neuron apoptotic process
learning
regulation of muscle system process
endothelial cell migration
regulation of axonogenesis
regulation of postsynaptic membrane potential
excitatory postsynaptic potential
0.03
0.04
0.05
0.06
GeneRatio
p.adjust
Count
B
AlzCode Platform
Aβ42 oligomers vs Vehicle
Random Genes
0.0001
380
303
number of interactions with AD associated genes
500
400
300
200
100
0
C
Interactions with disease genes
Density
200
250
300
350
400
Interactions
p.value<0.0001
D
AD vs Control
Aβ42 Oligomers vs vehicle
ACC
CBE
DLPFC
FP
IFG
PCC
PHG
STG
TCX
DEGs
1
0
-1
-2
Fold Change

Figure EV4. Aβ42 oligomers induce an RNA-signature present in AD-affected areas.

(**A**) The dotplot shows the significant Gene Ontology Biological processes that emerged from the enrichment analysis of DEGs in Aβ42 oligomers versus Vehicle, performed with ClusterProfiler. The name of the pathway is reported on the left of the graph, the *x*-axis represents the gene ratio, dots size represents the number of DEGs present in the pathway and the color indicates the adjusted *p* value of the pathway enrichment. Statistical analysis was performed with over-representation analysis for Gene Ontology Biological processes on DEGs, performed with ClusterProfiler (Wu et al, 2021). (**B**) The histogram shows the extent of correlation between DEG in Aβ42oligomers vs vehicle and known AD-associated genes when compared to random genes or known AD-associated genes. (**C**) Plot as generated by http://alzcode.xyz showing the number of the interactions of DEGs in Aβ42oligomers vs vehicle and known AD-associated genes (red line) compared to the distribution of interactions between random genes and known AD-associated genes. The significance is calculated by permutation test (Lin et al, 2022). (**D**) Heatmap showing the 477 DEGs in Aβ42 oligomers-treated vs vehicle-treated primary neurons and in human AD patients vs control patients in the indicated brain areas. ACC anterior cingulate cortex, CBE cerebellum, DLPFC dorsolateral prefrontal cortex, FP frontal pole, IFG inferior frontal gyrus, PCC posterior cingulate cortex, PHG parahippocaml gyrus, STG superior temporal gyrus, TCX temporal cortex.

 © The Author(s)

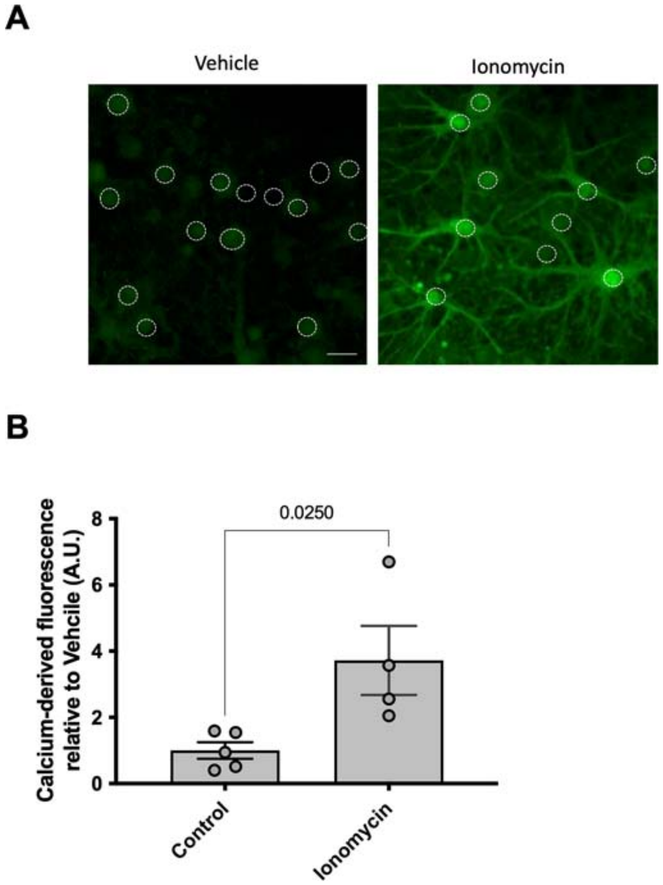

Figure EV5. Positive control for Ca^{2+} measurements in mouse primary neurons.

Treatment of mouse primary neurons with 2 μM ionomycin for 2 h was used as positive control for Ca^{2+}-derived fluorescence signal. Each circle represents an independent biological replicate. (**A**) Representative confocal microscopy images of mouse primary neurons incubated with Fluo-4 probe, green fluorescence is generated upon Ca^{2+} binding to the Fluo-4 probe. White dashed-line circles indicate nuclei of neurons. White dashed-line circles indicate the nuclei of neurons. Bar correspond to 20 μm. (**B**) Bar graph shows semiquantitative measures of intracellular Ca^{2+}-derived fluorescence. (control, $n = 5$; Ionomycin, $n = 4$) Unpaired t test was applied and data are represented as mean ± SEM.

© The Author(s)

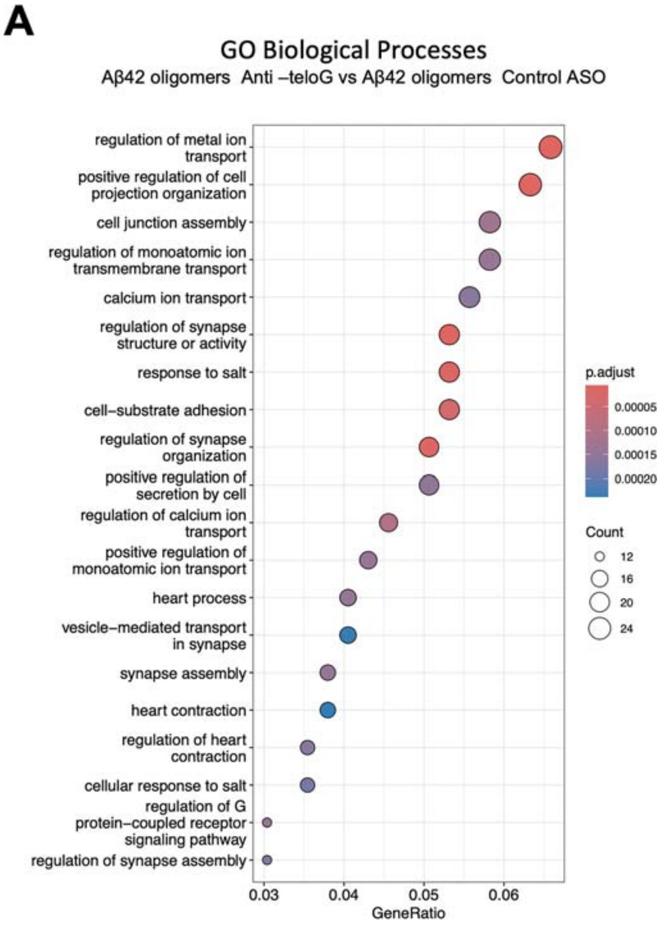

Figure EV6. tASO improves pathways dysregulated by Aβ42 oligomers.

(**A**) The dotplot shows the significant Gene Ontology Biological processes that emerged from the enrichment analysis of DEGs in Aβ42 oligomers Anti-Telo G-treated vs Aβ42 oligomers Control ASO-treated primary neuron, performed with ClusterProfiler. The name of the pathway is reported on the left of the graph, the *x*-axis represents the gene ratio, the dot size represents the number of differentially expressed genes present in the pathway and the color indicates the adjusted *p* value of the pathway enrichment. Statistical analysis was performed with over-representation analysis for Gene Ontology Biological processes on DEGs, performed with ClusterProfiler (Wu et al, 2021).

 © The Author(s)
